# Performance Evolution and Research Progress of Silicon Carbide Sensors in Radiation Environments: A Review

**DOI:** 10.3390/mi17070843

**Published:** 2026-07-16

**Authors:** Yan Liu, Yongxin Deng, Quanwei Zhang, Huafeng Li, Jue Wang, Yuan Wang, Fabin Cheng, Haijun Han, Peng Zhang

**Affiliations:** 1 Institute of Systems Engineering, China Academy of Engineering Physics, Mianyang 621900, China; liuyan123@caep.cn (Y.L.); sy2114205@buaa.edu.cn (Y.D.); swcaep@163.com (J.W.); orionwang@foxmail.com (Y.W.); cfb222@163.com (F.C.); hanhaijun@foxmail.com (H.H.); zhangp@caep.cn (P.Z.); 2 The State Key Laboratory for Manufacturing System Engineering, Xi’an Jiaotong University, Xi’an 710049, China

**Keywords:** SiC, radiation environment, sensing performance, radiation damage

## Abstract

Silicon carbide (SiC), a third-generation wide-bandgap semiconductor, demonstrates prominent application advantages for extreme-environment sensing scenarios including deep-space exploration, nuclear reactor monitoring, and fusion device diagnosis, which benefit from its excellent radiation resistance, high-temperature stability, and chemical inertness. This review systematically investigates the action mechanisms of different radiation environments on the electrical and mechanical properties of SiC-based sensors, with emphasis on the regulatory effects of radiation-induced defects on key sensing parameters, including piezoresistive properties, charge-collection efficiency, leakage current, and sensitivity. In addition, this paper discusses the response behavior and research progress of SiC sensors applied in mixed radiation fields. Existing research confirms that although high-fluence radiation can induce lattice defects and further result in the degradation of SiC sensor sensing performance, SiC still retains remarkable advantages in intrinsic radiation resistance. The sensing reliability of SiC in extreme environments can be further improved via device-structure optimization and material-modification strategies. This review is expected to provide a theoretical reference for the development and design of SiC sensors applied in advanced nuclear energy, aerospace, and nuclear medicine fields.

## 1. Introduction

Frontier fields, including deep-space exploration, advanced nuclear reactors, magnetic confinement fusion devices, and FLASH radiotherapy, impose extremely stringent requirements on sensing technologies. Sensor devices must operate stably over extended periods under extreme conditions such as high temperatures, intense radiation, and corrosive atmospheres. Silicon Carbide (SiC) has emerged as one of the most promising and well-studied wide-bandgap semiconductors for particle radiation detection, owing to its excellent material properties, including high radiation tolerance. Recent experimental results have demonstrated the successful application of SiC diodes for detecting electrons, protons, alpha particles, ions, UV radiation, X/γ-rays, and neutrons. However, the effects of high-temperature operation and radiation-induced damage on detector performance remain critical considerations for extreme-environment applications, where traditional silicon-based sensors are limited by their narrow bandgap and low displacement damage threshold [1].

Wide-bandgap semiconductors, particularly 4H-SiC and GaN-based devices, have exhibited exceptional performance in the detection of alpha particles and thermal neutrons. Among SiC polytypes, 4H-SiC possesses a 3.26 eV wide bandgap, a high critical breakdown electric field, high thermal conductivity, and excellent displacement damage resistance, making it one of the most promising candidate materials for radiation detection applications in extreme environments [2,3]. In recent years, research on SiC-based sensors under the synergistic extreme conditions of high temperature and irradiation has gradually emerged as a research hotspot. Existing studies have confirmed that SiC sensors can maintain stable piezoresistive responses at temperatures exceeding 500 °C, but the long-term reliability of such devices after accumulated radiation doses reach the MGy level still requires further experimental verification. This review summarizes current challenges and research gaps in the application of SiC sensors in extreme radiation environments and discusses future development directions for SiC-based sensing devices [4].

The influence of radiation environments on the performance of SiC sensors is twofold. The interaction between high-energy particles or photons and SiC crystal lattices induces the formation of point defects, defect clusters, and even amorphous regions. These defects function as carrier recombination centers or traps, altering the carrier transport properties of the material and, consequently, modifying the performance of electrically transduced SiC sensors—for example, changing the sensitivity of piezoresistive pressure sensors and the charge-collection efficiency (CCE) of ionization-type sensors [5]. Conversely, radiation-induced defects can also be exploited for specific sensing applications: for instance, measurable electrical signals can be obtained by utilizing nuclear reaction products in neutron detection [6,7]. The common types of radiation encountered in these application scenarios include charged particles (protons, alpha particles, heavy ions, with characteristic energies ranging from keV to GeV), neutrons (thermal neutrons with a wavelength of approximately 1.8 Å, fast neutrons with energies exceeding 1 MeV), and high-energy photons (X-rays and γ-rays, with wavelengths ranging from the picometer to nanometer scale). Different types of radiation interact with SiC lattices through distinct physical mechanisms. Accordingly, elucidating the evolution law of SiC sensing performance under different radiation types and irradiation conditions provides important guidance for the reliability design of SiC sensors deployed in radiation environments.

Based on research advances in recent years, this review organizes the relevant research progress from multiple dimensions. This work first analyzes the differential impacts of different radiation sources on the sensing performance of SiC semiconductors, which constitutes the core foundation of the overall analysis. On this basis, this review further clarifies the structure-activity relationship between radiation-induced defects and key sensing parameters, sorts out the application practices of SiC sensors in various typical radiation scenarios, summarizes the existing material and device engineering strategies for improving the radiation hardness of SiC sensors, and ultimately provides a reference for subsequent related research.

## 2. Influence of Radiation on the Electrical Sensing Performance of SiC

### 2.1. Correlation Between Radiation-Induced Defects and Carrier Transport Characteristics

Radiation-induced lattice defects are the fundamental intrinsic factors driving the degradation of the electrical sensing performance of SiC materials. Different types of radiation induce defects with markedly divergent characteristics in SiC in terms of category, concentration, and spatial distribution, which in turn produce distinct regulatory effects on carrier generation, recombination, and transport processes. The combined application of Technology Computer Aided Design (TCAD) numerical simulation and Deep-Level Transient Spectroscopy (DLTS) characterization offers an effective methodological approach for elucidating the quantitative correlation between radiation-induced defects and electrical sensing performance parameters of SiC.

**Figure 1:**
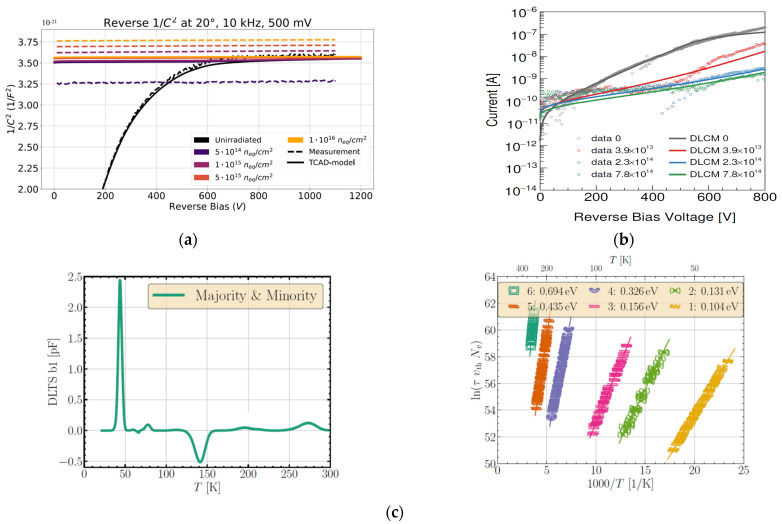
The correlation between defects and carrier transport properties in SiC: (**a**) Reverse bias 1/C 2-V measurements vs. simulation. Reprinted with permission from Ref. [5]. Copyright 2024 Elsevier. (**b**) Measured and simulated I-V by DLCM for diodes irradiated. Reprinted with permission from Ref. [11]. Copyright 2025 IOP Publishing. (**c**) Test results for deep-level transient spectroscopy of defects. Reprinted from Ref. [8].

A systematic TCAD modeling study by Gaggl et al. developed a bulk radiation-damage model for neutron-irradiated 4H-SiC pad diodes, optimized based on the existing literature and validated against experimental measurements. As shown in Figure 1a, the simulation successfully reproduced key experimentally observed effects, including capacitance flattening, loss of rectification properties, and CCE degradation. Importantly, the EH4 center was identified as a major lifetime killer in 4H-SiC, while the EH6,7 deep-level defect was suggested to be of donor type, which significantly influences carrier transport and recombination processes [5]. Using DLTS and Thermally Stimulated Currents (TSC), Sorgenfrei et al. quantitatively characterized electrically active defects in non-irradiated n-type epitaxial 4H-SiC diodes, determining defect parameters including concentration, activation energy, and capture cross-section, as shown in Figure 1c. The study identified the Z1/2 defect and a nitrogen-related defect as intrinsic defects present prior to irradiation, originating from vacancies, impurities, doping imperfections, or growth processes. These intrinsic defects provide a baseline understanding of how defects influence sensing performance stability, particularly at elevated temperatures where their impact becomes more pronounced. This study is conducted in the context of a radiation-hardness study of 4H SiC sensors and to further investigate present defects and ascertain their chemical structure, multiple irradiation campaigns are being carried out [8].

Burin et al. reported a simulation-based radiation damage modeling approach for 4H-SiC using TCAD tools under forward and reverse bias up to 1 kV. After validating the TCAD framework against silicon measurements, the model was used to approximate experimental data from neutron-irradiated 4H-SiC p-i-n particle detectors. This approach successfully predicts the temperature dependence of leakage current in SiC diodes after irradiation at different proton fluences and provides a feasible computational framework for lifetime prediction of SiC sensors in radiation environments. Importantly, the simulations offer an explanation for the almost negligible current of irradiated devices under high forward bias [9]. Meanwhile, molecular dynamics simulations carried out by Jiang et al. explored the influence of Shockley-type stacking faults (SSFs) on radiation displacement cascade effects in 4H-SiC. The simulations were conducted under different primary knock-on atom energies and temperature conditions, analyzing the variation pattern of radiation displacement defects and clusters. The results indicated that SSF limits defect distribution positions and affects both defects and clusters in the displacement cascade. Importantly, the faulted regions can significantly enhance defect recombination efficiency and partially mitigate the accumulation of radiation damage, while also influencing the maximum working temperature of 4H-SiC [10].

To further clarify the mechanisms of proton-induced defects, Li et al. systematically investigated the mechanisms of 80 MeV proton irradiation-induced defects on the electrical performance of 4H-SiC PiN sensors. Using DLTS and Time-Resolved Photoluminescence (TRPL), they analyzed defect characteristics and minority carrier lifetime before and after irradiation. Through current-voltage (I-V) and capacitance-voltage (C-V) measurements, it was observed that the sensor leakage current after proton irradiation decreased with increasing fluence, accompanied by a distinct trend, as shown in Figure 1b. A Deep-Level Compensation Model (DLCM) was established using the open-source TCAD simulation tool RASER to explain this phenomenon, as well as the constant-capacitance behavior exhibited under proton irradiation up to 7.8 × 10^14^ neq/cm^2^. More importantly, radiation-induced deep-level defects caused a certain degradation in the sensor’s energy resolution [11].

The response of neutron-irradiated 4H-SiC diodes to α-particles was examined by Gaggl et al. using 50-µm-thick 4H-SiC p-in-n planar pad sensors at room temperature with reverse biases up to 1100 V. The experimental results indicated that even at fluences reaching 10^15^ cm^−2^, the sensor could still resolve the α-particle peak, although the energy resolution deteriorated to some extent. Quantitative analysis showed a drop in CCE with increasing irradiation fluence: CCE values of 64% and 51% were obtained at fluences of 5 × 10^14^ neq/cm^2^ and 1 × 10^15^ neq/cm^2^, respectively, decreasing to 15% at 5 × 10^15^ neq/cm^2^. The study also noted that the sensor leakage current after irradiation increased nonlinearly with reverse bias voltage, a phenomenon directly linked to radiation-induced trap-assisted tunneling current. The measured CCE values agreed well with earlier UV-TCT studies, with discrepancies between 1% and 5% [12].

Heavy ion irradiation generates high-density electron-hole pairs while simultaneously inducing strong displacement damage, which is particularly destructive to SiC sensing performance. Comparative radiation damage studies by Altana et al. on large-area p-n junction SiC devices and standard silicon p-n junction detectors demonstrated that SiC devices exhibit radiation resistance more than two orders of magnitude higher than silicon devices. The SiC devices showed excellent performance in terms of parameter stability, linearity, defect distribution, CCE, energy resolution, and leakage current. The novel construction technology applied to SiC has enabled the creation of very robust devices with excellent performance, suitable for scientific projects requiring high resistance to radiation damage [13]. In a complementary study, Kucal et al. investigated temperature effects on radiation damage in 3C-SiC by irradiating single crystals with carbon and silicon ions at room temperature and at 800 °C. Using channeling Rutherford backscattering spectrometry, they found that the experimentally determined depth profiles of induced defects at room temperature agreed very well with theoretical calculations assuming proportionality to electronic and nuclear stopping power values. A significant reduction in crystal defects was observed for irradiations performed at high temperatures or for samples annealed after irradiation. Additionally, indications of saturation of crystal defect concentration were observed for higher fluences [14].

The impact of gamma-ray radiation on SiC sensing performance is mainly manifested in ionization damage and interface state generation. Feng et al. investigated the differential degradation of static and dynamic characteristics of SiC Metal-Oxide-Semiconductor Field-Effect Transistors (MOSFETs) under gamma irradiation. Total ionizing dose (TID) irradiation caused a threshold voltage shift (toward negative due to radiation-induced positive oxide-trapped charges) and switching response degradation, with turn-off time being more sensitive to TID irradiation compared to turn-on time. Sentaurus TCAD 2-D simulations verified that high-speed accumulation of gate oxide traps in the localized region of the trench-gate MOS due to non-uniform electric field distribution is the main reason for the special degradation of electrical parameters under drain bias [15]. Li et al. investigated the impact of gamma irradiation and subsequent room-temperature annealing on commercial 4H-SiC Schottky barrier diodes (SBDs). The ideality factor increased from 1.01 to 1.13 with increasing irradiation dose but recovered after 7 days of room-temperature annealing. Current-voltage and capacitance-voltage measurements showed that Schottky barrier heights varied little with irradiation dose and recovered after 7 days. DLTS analysis revealed the presence of Z1/Z2 traps after annealing, with activation energies ranging from 0.46 eV to 0.55 eV, and trap concentration increased from 9.48 × 10^12^ cm^−3^ to 2.23 × 10^13^ cm^−3^ with increasing irradiation dose. These findings indicate that gamma irradiation-induced point defects were the primary cause of degradation in 4H-SiC SBDs [16]. Metreveli et al. investigated gamma irradiation effects on 4H-SiC bipolar junction transistors (BJTs) under different biasing regimes (saturation, cut-off, active, reverse, and zero bias) using three different dose rates. In situ characterization was performed to avoid delays between irradiation and measurement. The experiments demonstrated that 4H-SiC bipolar transistors can withstand high gamma doses, with less than 22% degradation of current gain at doses up to 2 Mrad(Si) under the worst-case conditions [17]. Furthermore, Metreveli et al. presented a Simulation Program with Integrated Circuits Emphasis (SPICE) model for 4H-SiC BJT and TTL inverters exposed to gamma radiation up to 800 krad(Si). The adjusted VBIC-based SPICE model accounts for both bulk and surface degradation mechanisms by extracting parameters of forward current gain, saturation current, base resistance, and forward transit time. Results show uniform degradation of BJTs primarily manifested as reduced current gain and increased base resistance, while the inverter maintained functional operation up to 600 krad(Si). Extrapolation of the SPICE model predicts a failure threshold near 16 Mrad(Si), far exceeding the tolerance of conventional silicon circuits. By linking radiation-induced defects at the material and interface levels to circuit-level behavior, the proposed model enables realistic design and lifetime prediction of SiC integrated circuits for radiation-intensive applications [18].

The displacement-damage capability of electron irradiation is weaker than that of neutrons or heavy ions, but high fluences can still cause significant sensing performance degradation. Niskanen et al. investigated the effect of 20 MeV electron radiation on the lifetime of SiC power MOSFETs by applying accelerated constant-voltage stress (CVS) on pristine and irradiated devices. The time-to-breakdown and charge-to-breakdown of gate oxide were extracted and compared. The effect of electron radiation on device lifetime reduction was observable at lower stress gate-to-source voltage levels. The authors proposed models of TBD and QBD dependence on initial gate current to describe device breakdown behavior [19]. With regard to radiation swelling, Kobayashi and Alam introduced a physics-regularized neural network (PRNN) as a computational approach to predict SiC swelling under irradiation, particularly at high temperatures. The PRNN model combines physics-based regularization with neural network methodologies to generalize SiC behavior even in conditions beyond traditional empirical model valid ranges. Using nested cross-validation to ensure robustness and generalizability, the PRNN effectively bridges empirical and sparse experimental data by integrating prior knowledge and refined tuning procedures, demonstrating predictive power in high-irradiation conditions essential for nuclear and aerospace applications [20].

Advances in defect characterization techniques have further deepened the understanding of the structure–activity relationship of radiation-induced defects. Mokhov et al. conducted a comprehensive study of intrinsic defects in sublimation-grown SiC crystals as a function of growth conditions and thermal annealing. Complexes of intrinsic defects, including carbon vacancies and impurity atoms, were found in Si-rich SiC crystals grown by physical vapor transport at low temperatures below 2200 °C. Intrinsic defects in grown SiC crystals are characterized by high thermal stability (up to 2600 °C), associated with the presence of active metastable clusters. Paramagnetic defects in SiC are considered a material platform for sensing, quantum photonics, and information processing at ambient conditions [21]. Coutinho presented a theoretical study of the electronic and dynamic properties of silicon vacancies and self-interstitials in 4H-SiC using hybrid density-functional methods. The silicon site vacancy and the carbon-related antisite-vacancy (CAV) pair are interpreted as a unique and bistable defect. The vacancy introduces a (−/+) transition calculated at E_c_-1.25 eV, which determines a temperature threshold for VSi annealing into CAV in n-type material. VSi anneals out in two stages: at low temperatures (≲600 °C) via capture of mobile species (e.g., self-interstitials) and at higher temperatures (≳1200 °C) via dissociation into VC and CSi defects. The silicon interstitial is also a negative-U defect with metastable q = +1 and q = +3 states, which may explain why it has escaped detection [22]. Pellegrino et al. irradiated 4H-SiC p-n junctions with 700 keV He+ ions over a fluence range of 1.0 × 10^12^ to 1.0 × 10^15^ ions/cm^2^. Two fluence regimes were identified: at low fluences (≤10^13^ ions/cm^2^), I-V characteristics showed increased series resistance associated with decreased dopant concentration, and the main defect states produced were Z1/2, RD1/2, and EH6/7 centers. At high fluences (>10^13^ ions/cm^2^), I-V curves showed a strong decrease in generation current, and DLTS evidenced defect rearrangement. The results suggest the formation of localized highly resistive regions (by agglomeration of point defects) in parallel with the main junction [23].

### 2.2. Differential Effects of Different Radiation Types on Sensor Charge-Collection Efficiency

SiC radiation sensors function by collecting electron–hole pairs generated by incident radiation within the depletion region of Schottky junctions or p–n junctions. High-energy radiation induces two primary damage mechanisms in SiC: (1) ionization damage, which generates electron–hole pairs that can be trapped in oxide layers or at material interfaces; (2) displacement damage, in which energetic particles (including neutrons, protons, and ions) displace host atoms from their original lattice positions, resulting in the formation of point defects (vacancies and interstitials) and defect clusters. These defects act as recombination centers or charge-trapping sites, leading to a reduction in CCE, an increase in leakage current, and changes to piezoresistive coefficients.

CCE is a core parameter for evaluating the sensing performance of SiC radiation sensors, directly determining the energy resolution and detection sensitivity for incident particles. The interaction mechanisms between different radiation types and SiC differ, causing the degradation behavior of CCE to exhibit significant energy dependence and fluence threshold characteristics. In fast neutron irradiation scenarios such as nuclear reactor core monitoring, the evolution of CCE in 4H-SiC Schottky barrier sensors has been systematically reviewed by Ruddy et al., who summarized the effects of fast neutrons (E > 1 MeV) at low fluences (<10^12^ cm^−2^) and higher fluences (up to 10^17^ cm^−2^). The wide bandgap of SiC (3.27 eV) allows operation at temperatures up to 700 °C and probably higher, unlike conventional low-bandgap semiconductors (silicon, germanium), which are limited by thermally generated charge carriers. It was summarized that, with increasing fast neutron fluence, there are two phases: a defect cluster formation phase and a carrier transport channel obstruction phase. The effects of irradiation temperature on the accumulation of radiation damage are also discussed [24].

**Figure 2:**
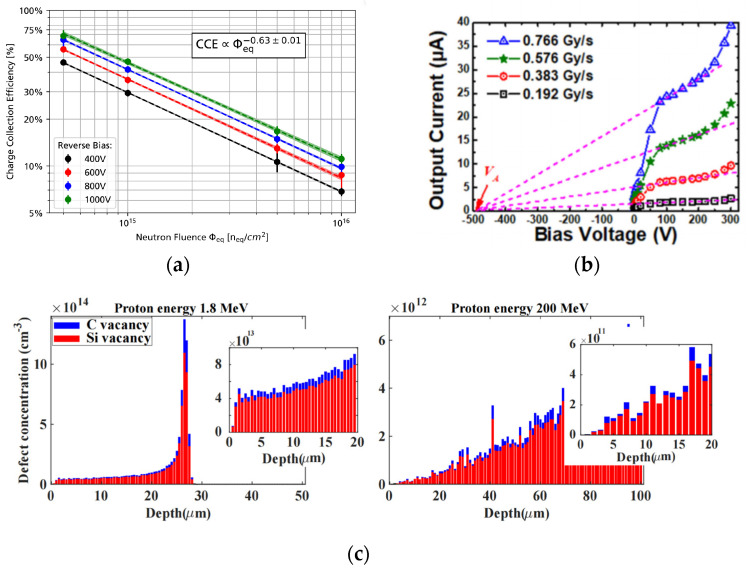
Charge-collection efficiency (CCE) degradation characteristics of 4H-SiC detectors under different Radiation: (**a**) Power-law degradation curve of CCE with 1 MeV equivalent neutron fluence for 4H-SiC PiN diode. Reprinted with permission from Ref. [25]. Copyright 2023 IOP Publishing. (**b**) The extraction of the early voltage V_A_ of the SiC NPN detector under X-ray illumination. Reprinted from Ref. [35]. (**c**) Defect concentration depth distribution of 4H-SiC under 1.8 MeV and 200 MeV proton irradiation. Reprinted with permission from Ref. [30]. Copyright 2023 IOP Publishing.

Spatially resolved CCE characterization of neutron-irradiated 4H-SiC PiN diodes was further performed using the Ultraviolet Transient Current Technique (UV-TCT) by Gsponer et al. The study covered neutron fluences up to 1 × 10^16^ neq/cm^2^, with leakage currents remaining extremely small (below 10 pA at 1.1 kV reverse bias). A pronounced CCE reduction region was observed at the edge of the sensor active area after irradiation, a phenomenon directly related to irradiation-induced surface defects, indicating that greater attention should be paid to the radiation-hardness optimization of edge termination structures. Interestingly, the study also found that CCE in forward bias was increased relative to reverse bias, as shown in Figure 2a, and CCE surpassing 100% was observed in alpha and UV-TCT measurements, requiring further systematic investigation [25]. By means of the same UV-TCT, Gaggl et al. quantitatively measured the depth distribution of CCE in neutron-irradiated 4H-SiC p-on-n planar diodes. The study covered fluences from 5 × 10^14^ to 1 × 10^16^ neq/cm^2^, with dark current levels remaining in the nA range for all fluences. The results revealed that CCE degradation was more severe near the surface, consistent with the shorter range of recoil atoms generated by neutron collisions with SiC. It was also found that the decrease in CCE with irradiation fluence could be partially compensated when operating the samples at reverse-voltage conditions far above full depletion [26].

Thermal neutron detection typically requires a conversion layer, such as ^6^LiF or ^10^B, on or inside the SiC, making use of nuclear reaction products to generate measurable signals. Comparative irradiation experiments on SiC p-n sensors of different structures were conducted at the RA-6 research reactor by Pérez et al. [27]. For thermal neutron measurements, 50 µm and 100 µm SiC diodes coupled to a ^6^LiF conversion layer were tested, exhibiting a linear response with reactor power up to 500 kW with no saturation or dead-time effects, and an intrinsic detection efficiency of (4.39 ± 0.22)%. It was found that the radiation stability of the conversion layer/SiC interface is the critical factor determining long-term reliability. After high-fluence thermal neutron irradiation, sensors with a ^6^LiF conversion layer suffered interface delamination and a distinct drop in detection efficiency; in contrast, integrated sensors fabricated by ^10^B implantation showed much smaller efficiency losses under the same fluence. For fast neutron detection, 100 µm diodes coupled to polypropylene layers achieved a maximum intrinsic detection efficiency of (0.57 ± 0.04)% for an 800 µm layer. PHITS Monte Carlo simulations reproduced the experimental data, validating the interpretation of the measured spectra. These results confirm that, for long-term in-core neutron monitoring, the integrated design offers superior reliability over thin-film coating approaches. Subsequent comprehensive characterization of the novel SiC neutron sensors by Pérez et al. verified their discrimination capability in mixed fields. The device is based on a 50-µm-thick p-n diode fabricated on a 4H-SiC wafer. Using enriched LiF conversion layers, a detection efficiency of 6 ± 1% was achieved with a 25-µm-thick LiF layer for thermal neutron detection. The detector was also confirmed to be capable of detecting recoil nuclei and protons produced by fast neutrons. Experimental spectra were compared with PHITS simulations, validating the detector’s performance for potential applications in various scientific and technological fields [28]. A trench Schottky-type 4H-SiC neutron sensor was developed by Jiang et al., in which the neutron conversion material ^6^LiF was filled into the trench array to significantly enhance the thermal neutron capture probability. By etching trenches, both the amount of ^6^LiF filling and the total contact area between ^6^LiF and 4H-SiC were increased. The detector achieved a count rate as high as 580 Hz with a detection efficiency of 6.57%, breaking through the limitation of thin-film coated-type detectors (typically less than 5%). The use of refractory metal tungsten (W) as the front Schottky contact electrode enabled the detector to maintain a count rate of approximately 100 Hz at 180 °C, demonstrating the possibility of high-temperature operation with extremely low gamma-ray sensitivity [29].

The effect of proton irradiation on the CCE of SiC sensors is strongly energy dependent. A comparison of the electrical properties of 4H-SiC Schottky diodes after irradiation with protons of different energies by Kumar et al. showed that low-energy protons (1.8 MeV), because of their higher nuclear stopping power, create a high concentration of defects near the SiC surface, causing the energy resolution for α-particles to degrade significantly at a fluence of only 10^13^ cm^−2^. In contrast, high-energy protons (200 MeV) are dominated by electronic stopping, with displacement damage concentrated mainly at the end of the particle track, as shown in Figure 2c, so the overall CCE degrades more slowly. Post-irradiation improvements in ideality factors, reductions in threshold voltage, and increases in breakdown voltage were observed for high-energy proton irradiation, with point defects likely responsible. Consecutive DLTS measurements after reverse-bias anneals from 350–700 K explored the annealing behavior of proton-induced defects [30]. In contrast, high-energy protons are dominated by electronic stopping, with displacement damage concentrated mainly at the end of the particle track, so the overall CCE degrades more slowly. Research on proton-irradiated 4H-SiC Low-Gain Avalanche Detectors (LGADs) by Satapathy et al. using 2.5 GeV protons at fluences up to 3.33 × 10^14^ p/cm^2^ revealed severe performance degradation. At the highest proton fluence, LGADs displayed a loss of rectification, high ON-state resistances (>10^10^ Ω-cm^2^), and the complete removal of gain. Reductions in capacitance and OFF-state current pointed to compensation of the gain layer as the gain-reducing mechanism. Radiation-induced defects also hindered carrier acceleration, reducing impact ionization and further gain reduction. However, LGADs exposed to a fluence of 1 × 10^14^ p/cm^2^ experienced a partial recovery in gain (from the original ∼2 to ∼1.65 at 500 V bias). Despite the reduction in device performance, the demonstration of a measurable signal after irradiation points to the potential of SiC LGADs for future high-energy physics applications [31].

Mixed and high-dose-rate radiation fields are encountered in both space and medical applications, requiring the synergistic effects of multiple particles to be considered. In situ radiation damage studies on SiC sensors placed in a 252.7 MeV proton therapy beam were conducted by Radmanovac et al. Planar PiN diodes from two different manufacturers were exposed to increasing fluences ranging from 1.4 × 10^11^ to 3.5 × 10^13^ p^+^/cm^2^. Electrical characterizations performed in situ between fluence steps revealed a gradual compensation of the effective epitaxial doping concentration with each incremental fluence step, observed as a reduction in capacitance before full depletion. Linear donor removal rates were determined for all sample groups, with values ranging from 4.2 cm^−1^ to 6.4 cm^−1^. These findings provide a quantitative basis for understanding radiation-induced charge carrier removal in 4H-SiC devices and are relevant for predicting the performance and lifetime of future radiation-hard detector technologies [32]. Novel SiC sensors were applied for the first time by Romano et al. to characterize ultra-high-dose-rate (UHDR) electron beams for FLASH radiotherapy. The study investigated the response of a newly developed 1 × 1 cm^2^ SiC sensor as a function of dose-per-pulse and its radiation hardness up to a total delivered dose of 90 kGy using UHDR pulsed electron beams accelerated by a dedicated ElectronFLASH LINAC. Results showed a linear response up to 2 Gy/pulse and a variation in charge per pulse within ±0.75% for a cumulative delivered dose of 90 kGy. The sensor demonstrated the capability to maintain sub-nanosecond time resolution even at extremely high instantaneous dose rates, representing the first characterization of SiC with UHDR-pulsed beams from a dedicated ElectronFLASH LINAC [33].

For X-ray and γ-ray detection, SiC sensors also exhibit excellent radiation resistance. Characterization of several SiC sensor technologies using synchrotron radiation X-rays by Paz et al. indicated that the detection efficiency for X-rays below 30 keV is mainly limited by the active region thickness. The photodiodes exhibited over-response values of 12–19% under 20 keV X-ray irradiation, which were attributed to interactions between the radiation and the metallic layers. Good response uniformity and linearity were observed at 0 V bias. Importantly, the degradation in energy resolution after high cumulative doses remained at a relatively low level. This work exemplifies the good performance of SiC detectors fabricated at IMB-CNM specifically for low-energy X-ray characterization at high X-ray intensities [34]. A SiC NPN-structure radiation sensor was developed by Wang et al. that shows outstanding response sensitivity to X-rays (up to 30 keV photon energy) with extremely low dark current density (less than 1 nA). The sensitive region of the radiation detector is much thicker (30 μm) compared to traditional photodetectors, ensuring high energy deposition of radiation particles. Due to the fully depleted sensitive region and bipolar transistor configuration, the SiC NPN detector exhibited a clear common-emitter current gain of 5.85 at 200 V (under 0.383 Gy/s), as shown in Figure 2b, with the gain increasing with bias voltage due to the Early effect and reaching 7.55 at 300 V. The NPN detector with internal gain showed great potential in radiation detection [35]. Transient behavior of SiC α-particle sensors was analyzed by He et al. using SRIM-informed TCAD simulation. The study identified the external bias voltage and incident particle energy as key factors influencing transient current pulse broadening. Low-energy alpha particles result in low initial kinetic energy of ionization-generated carriers, leading to transient current broadening and reduced time resolution. Conversely, high-energy alpha particles ionize carriers with high drift velocities, preventing the broadening effect. The simulation results agreed well with experiments, confirming the reliability of TCAD tools for predicting sensor performance and providing valuable guidance for optimizing alpha particle detectors, selecting appropriate bias voltages, and enhancing time-resolution capabilities [36].

### 2.3. Influence of Radiation on the Piezoresistive Effect in SiC

The piezoresistive effect constitutes the fundamental physical principle underlying SiC-based mechanical sensing devices. Stress-induced modulation of the energy band structure leads to variations in the effective mass and mobility of charge carriers, which ultimately give rise to the piezoresistive response. Lattice defects introduced by irradiation alter the energy band structure and carrier scattering processes in SiC, thereby significantly changing the piezoresistive coefficient and the sensitivity of SiC-based sensors.

Under non-irradiated conditions, the intrinsic piezoresistive properties of SiC have been systematically explored. An all-SiC fiber-optic Fabry–Perot (FP) pressure sensor fabricated by hydrophilic direct bonding technology was demonstrated by Liang et al., achieving stable pressure measurement at 600 °C. The FP cavity is formed by hermetically direct bonding of two SiC wafers, including a thinned SiC diaphragm and a SiC wafer with an etched cavity. White-light interference is used for the detection and demodulation of pressure signals. Experimental results demonstrated sensing capabilities for pressure up to 800 kPa. The all-SiC structure without any intermediate layer can avoid sensor failure caused by thermal expansion coefficient mismatch, showing great potential for pressure measurement in high-temperature environments [37]. Li et al. studied the stability of electrical connections in high-temperature pressure sensors based on the piezoresistive effect of p-type SiC. A varistor with a positive trapezoidal shape was designed and etched innovatively to improve contact stability between the metal and the SiC varistor. Excellent ohmic contact was formed by annealing at 950 °C between Ni/Al/Ni/Au and p-type SiC with a doping concentration of 10^18^ cm^−3^. Aging tests in air from 25 °C to 600 °C showed that varistor resistance initially decreased, then increased with temperature, reaching a minimum at ~450 °C. It was found that the contact resistance increased by about 15% after aging at 500 °C for 100 h. The maximum temperature coefficient of resistance (TCR) of about −0.35%/°C was observed at ~100 °C. The encapsulated sensor exhibited output sensitivity of approximately 1.09 mV/V/bar at room temperature [38].

A 4H-SiC piezoresistive accelerometer manufactured using femtosecond laser and MEMS processing technology was demonstrated by Yang et al., verifying its potential for high-temperature applications. A femtosecond laser was adopted to reduce the cantilever thickness and release the proof mass, overcoming the SiC deep etching problem. The designed sensor exhibited a sensitivity of 0.0763 mV/g/5 V for low-g vibration tests with a resonant frequency of 1145 Hz. High-temperature performance was verified: at 250 °C, sensitivity was 0.0529 mV/g/5 V, with a sensitivity temperature coefficient of −0.12% FSO/°C and a zero-offset temperature coefficient of −1.38% FSO/°C. The research verifies the feasibility of femtosecond laser etching for SiC accelerometers and the high-temperature working ability of SiC accelerometers [39]. Fang et al. fabricated platinum (Pt) thin-film resistance temperature detectors (RTDs) on SiC substrates using Al_2_O_3_ as a transition layer and AlN grooves for alignment. The composite layers strongly adhered to the substrate at temperatures reaching 950 °C, and the Al_2_O_3_/Pt bilayer interface remained stable at elevated temperatures around 950 °C. This stability contributed to excellent high-temperature electrical performance, enabling the Pt RTD to endure temperatures exceeding 850 °C with good linearity. Through interface optimization, a matching Pt thin-film resistance temperature sensor maintained a contact resistance change of less than 5% after aging at 500 °C. The study also inferred that tensile stress and self-diffusion of Pt films lead to hillock formation, ultimately reducing electrical performance [40]. These studies indicate that key performance metrics, including the piezoresistive coefficient, linearity, contact stability, and processing quality, are strongly influenced by material- and device-level factors, including crystal orientation, bonding processes, electrode materials, and processing-induced defects.

**Figure 3:**
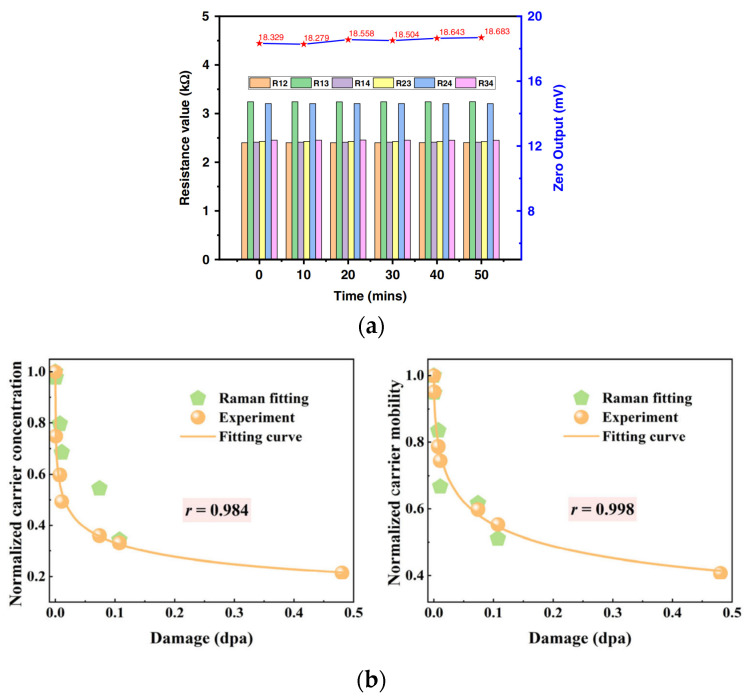
Influence of radiation damage on the electrical properties and sensing performance of SiC: (**a**) The resistance value and zero output change after X-ray irradiation [41]. (**b**) Normalized carrier concentration and mobility of 4H-SiC as a function of ion-irradiation damage. Reprinted from Ref. [42].

Wu et al. systematically explored the nonlinear piezoresistive effect of 4H-SiC and developed a MEMS pressure sensor operating stably from −50 to 300 °C. Four-point bending experiments combined with band calculations revealed the crystallographic orientation dependence of the 4H-SiC piezoresistive coefficient. The TCR values of 4H-SiC piezoresistors were obtained from −50 to 500 °C, and a conductivity variation model based on scattering theory was established to reveal the nonlinear variation mechanism. The fabricated sensor showed good output sensitivity (3.38 mV/V/MPa), accuracy (0.56% FS), and low temperature coefficient of sensitivity (−0.067% FS/°C). Its radiation tolerance was confirmed by 5W X-ray irradiation experiments, as shown in Figure 3a, and survivability in extreme environments was further demonstrated by anti-corrosion capability in H_2_SO_4_ and NaOH solutions [41].

Raman spectroscopy characterization of ion-irradiated 4H-SiC by Dai et al. showed that the broadening and redshift of the E_2_(TO) characteristic peak were linearly correlated with the degradation of the piezoresistive coefficient, directly confirming the deteriorating effect of radiation damage on piezoresistive properties. A fine analysis of the A_1_(LO) phonon mode demonstrated that the proliferation of irradiation-induced acceptor centers and the accumulation of scattering defects lead to significant attenuation of carrier concentration and mobility, as cross-verified by Hall effect measurements, thereby causing a degradation in electrical conductivity. The study established a quantitative correlation model between conductivity degradation and total disorder quantified by the DI/DS model. This non-destructive Raman technique enables simultaneous acquisition of material-damage characteristics and quantitative electrical-performance degradation, as shown in Figure 3b, suggesting that Raman spectroscopy can serve as an effective in situ monitoring tool for radiation damage in SiC mechanical sensing elements [42]. A systematic investigation by Almaz and Blue examined the degradation of 4H-SiC piezoresistive pressure sensors under high-fluence fast-neutron and γ-ray mixed irradiation, as well as the effects of annealing at temperatures up to 400 °C. The results demonstrated that high-fluence neutron irradiation induced severe displacement damage in the SiC lattice, causing significant attenuation of the pressure-output voltage signal and even sensor failure. The observed carrier-removal effect induced by displacement damage alters the conductivity of the piezoresistive material. Pressure-output voltage results showed recovery after annealing, with bridge resistances remaining at the same level for annealing temperatures up to 300 °C. However, after annealing at 400 °C, resistance values changed dramatically, indicating that while annealing can partially repair irradiation damage, limitations remain for the complete recovery of piezoresistive performance [43].

Beyond these, SiC has also demonstrated radiation-tolerant operation potential. Zhang et al. constructed a self-powered SiC ultraviolet photodetector with wide applicability and high commercialization potential. Using the pyro-phototronic effect in N-doped 4H-SiC single-crystal photodetectors, a fast pyroelectric response time of 0.27 s was achieved, which is almost ten times shorter than that obtained from steady-state signals under UV illumination. This work reveals fundamental optoelectronic physics in SiC and highlights its potential for photosensing applications, including radiation-tolerant operation in optoelectronics [44]. Tsutsumi et al. developed 4H-SiC photosensors with active pixel sensor (APS)-type circuits for radiation-hardened CMOS image sensors. The photosensors with APS-type circuits showed high responses to UV light, demonstrating their operation for ultraviolet imaging applications. Importantly, after 2 MGy (SiO_2_) gamma-ray irradiation, the dark current was 25 nA/cm^2^, and the APS-type photosensors were successfully working, demonstrating MGy-class radiation hardness [45]. Yang et al. presented a position-sensitive photodetector (PSD) based on undoped 4H-SiC with a simple vertical structure that eliminates the need for complex multi-interface architectures. For optoelectronic position detection applications, it exhibited remarkable stability under gamma-ray irradiation (300 krad) with minimal photocurrent variation, making it suitable for accurate position tracking in radiation-prone environments [46]. Okeil and Wachutka presented an in-depth investigation of the magnetic-field-sensing capabilities of a 4H-SiC-based lateral pin diode up to 500 °C. The diode was characterized for magnetic-field sensitivity, linearity, stability, and current noise. By combining sensitivity and noise measurements, the magnetic field detectivity was evaluated. The results reflect the potential of the diode for magnetic field sensing in harsh environments as well as for electric current sensing applications in SiC power modules, demonstrating the radiation-tolerant operation potential of SiC in magnetic sensing applications [47]. Sgrignuoli et al. demonstrated the use of near-zero-field magnetoresistance (NZFMR) in SiC diodes for high-temperature relative magnetometry, achieving sensitive detection of weak magnetic fields at temperatures up to 500 °C. A critical advantage is the sensor head’s low power consumption of less than 0.5 W at 500 °C for magnetic fields below 5 Gauss, demonstrating the radiation-tolerant operation potential of SiC in magnetic sensing applications [48]. Khudher and Abd prepared pure and Ag-doped SiC films on p-type silicon wafers using pulsed laser deposition with different dopant ratios. The results demonstrate the potential of SiC-based gas sensors for radiation-tolerant operation [49]. Mamun et al. reviewed SiC-based DNA sensing technologies, highlighting that as a wide-bandgap semiconductor with excellent chemical, physical, electrical, and biocompatible properties, SiC is a promising material for DNA sensors. These technologies demonstrate the potential of SiC for biosensing applications requiring radiation-tolerant operation in harsh environments [50].

### 2.4. Enhanced Effects of Radiation Damage Under High-Temperature Synergy

In practical engineering applications such as nuclear energy systems and deep-space exploration, radiation environments typically coexist with high-temperature conditions. The synergistic degradation effect of these two factors on the sensing performance of SiC has emerged as a current research hotspot. High-temperature conditions not only modify the annealing behavior of radiation-induced defects but may also activate new evolution pathways for defects, thereby increasing the complexity of corresponding material failure modes.

**Figure 4:**
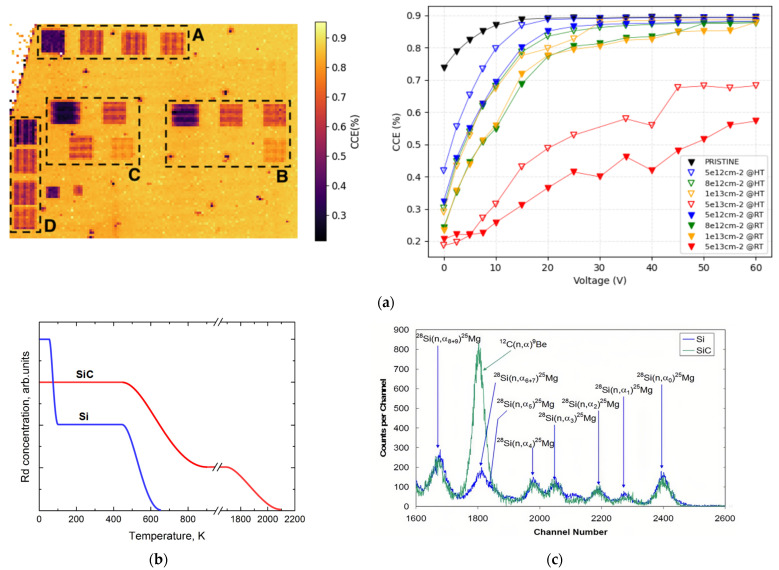
Research on Radiation Damage and Performance Response of SiC Sensors Under High-Temperature Irradiation: (**a**) IBIC mapping and CCE variation of SiC membrane sensors under room-temperature and 500 °C proton irradiation. Reprinted from Ref. [51]. (**b**) Schematic comparison of radiation defect annealing behavior in SiC and Si. Reprinted from Ref. [53]. (**c**) Enlarged view of neutron-induced reaction peaks in 4H-SiC detectors applied to fusion reactor environments. Reprinted from Ref. [55].

In situ proton-beam irradiation of SiC sensors at high temperatures was investigated by Medina et al. using an ion microprobe chamber (Figure 4a), which allowed exposure of small areas within the same device to different ion beams. The sensors tested were PiN diodes with ultrathin free-standing membranes realized by doping-selective electrochemical etching. The study reported changes in charge-transport properties, specifically CCE, with respect to multiple localized proton irradiations performed at both room temperature and 500 °C. The results revealed that the influence of temperature on radiation damage is nonmonotonic, with complex behavior that depends on the interplay between defect annealing and clustering processes [51]. Remy et al. fabricated 4H-SiC SBDs and evaluated their electrical parameters using I-V and C-V measurements at elevated temperatures up to 500 °C in 100 °C increments. Electrical performance was also measured at room temperature following each thermal cycle, along with energy-spectroscopy performance. The study successfully described the I-V characteristics by combining thermionic emission and field emission models. At low temperatures, the TFE model did not match experimental results well; however, a model combining TFE current and reverse saturation current (FE model) at room temperature proved a good fit with experimental data from room temperature to 500 °C when high bias was applied, providing theoretical guidance for the design of high-temperature radiation sensors [52].

Lebedev et al. systematically compared electron and proton irradiation effects on SiC at high temperatures (300–500 °C) and at room temperature, finding that the radiation resistance of SiC was significantly enhanced under high-temperature irradiation, as shown in Figure 4b. The main mechanism of SiC compensation is the formation of deep acceptor levels. With increasing irradiation temperature, the probability of formation of these centers decreases, and they are partly annealed out. As a result, the carrier removal rate in SiC becomes approximately six orders of magnitude lower for irradiation at 500 °C compared to room temperature. This effect results from the rapid annealing and recombination of point defects at high temperatures, which reduces the accumulation of stable defects. However, the types of residual defects differed: fewer vacancy-type defects and more antisite defects were observed after high-temperature irradiation, which may have different consequences for long-term stability compared with low-temperature irradiation. This once again proves that SiC is promising as a material for high-temperature electronic devices [53].

Further investigation by Lebedev et al. into the temperature dependence of SiC radiation resistance studied the effect of high-temperature electron and proton irradiation on the characteristics of commercial 4H-SiC integrated Schottky diodes with blocking voltages of 600, 1200, and 1700 V. It was found that the radiation resistance of SiC Schottky diodes under high-temperature irradiation significantly exceeds that under room-temperature irradiation, due to annealing of compensating radiation defects. The spectrum of radiation-induced defects introduced into SiC under high-temperature irradiation differed significantly from that introduced at room temperature. A comparison of radiation resistance between silicon and SiC showed that the relatively small difference in carrier removal rates at room temperature is because, unlike Si, there is practically no annealing of primary radiation defects in SiC during irradiation. Furthermore, the ranking of radiation tolerance among different SiC polytypes can change at high temperatures [54].

In fusion devices, SiC diagnostic window materials must simultaneously withstand 14 MeV neutron irradiation and temperatures exceeding 500 °C. As shown in Figure 4c, A review of SiC sensor progress for ITER and DEMO fusion reactors by Ruddy et al. highlighted that 4H-SiC Schottky barrier and p-n diode detectors are compatible with radiation monitoring applications in both fission and fusion reactors. The wide bandgap of 4H-SiC (3.27 eV) allows measurements at temperatures up to 800 °C and probably higher. The primary performance degradation originates from neutron-induced lattice expansion and thermal conductivity reduction. When neutron fluence reaches high levels, the thermal conductivity of SiC drops from ~350 W/(m·K) to below 100 W/(m·K), exacerbating Joule heat accumulation and forming a ‘radiation-thermal-electrical’ positive feedback failure mechanism. Importantly, 4H-SiC detectors have been demonstrated to provide linear count-rate responses for D-D and D-T fusion neutrons, and detectors with lithium neutron convertor foils can be used to directly measure tritium breeding rates in fusion reactors [55].

Grome and Hossain investigated the thermal expansion coefficient and amorphization in 4H-SiC containing point defects at different concentrations using classical molecular dynamics simulations. The results showed that 4H-SiC with vacancy defects exhibits a negative thermal expansion coefficient (TEC) above a critical defect density of around 9% (irrespective of temperature). With interstitial defects, it exhibits a positive TEC (regardless of defect density), and with Frenkel pair defects, it shows a transition from positive to negative TEC for defect densities greater than 8%. Negative thermal expansion in the 300–700 K range was observed in defective 4H-SiC after high-fluence irradiation. This anomalous thermodynamic behavior could potentially lead to thermal mismatch failure at the sensor packaging interface. The coupling between temperature-induced expansion and defect-induced stress in the lattice forms the mechanistic basis for the observed variation in TEC [56].

Beyond high temperatures, other harsh conditions, such as corrosive atmospheres and mechanical vibration, can also be coupled with radiation damage. Thermal simulation-based optimization of a novel SiC sensor design for neutron measurement at the JSI research reactor was performed by Valero et al. using COMSOL Multiphysics. The study targeted nuclear heating conditions corresponding to a TRIGA nuclear research reactor. By determining the temperature field for different configurations, the influence of various parameters, including housing material nature, gas nature around the diode, gas-gap height, and housing thickness, was analyzed. An improved thermal management scheme was shown to limit the sensor temperature rise in the core to within 50 °C, significantly extending its lifetime [57]. Cayley et al. discussed the challenges faced by silicon-based detector systems for dosimetry in radiation therapy, particularly considering emerging modalities such as ultra-high-dose-rate FLASH radiotherapy and mixed radiation fields (e.g., proton therapy). These challenges create exciting opportunities for new radiation detector materials, including SiC. While discussing novel materials for radiotherapy dosimetry, including SiC, the review pointed out that signal readout electronics in high-temperature radiation environments remain a current technical weakness that needs to be addressed for the successful implementation of SiC-based dosimetry systems [58].

## 3. SiC Sensor Applications for Specific Radiation Scenarios

### 3.1. SiC Sensors in Deep-Space Exploration

In deep-space exploration missions, spacecraft and the carried payloads are confronted with multi-source radiation hazards originating from galactic cosmic rays, solar particle events, and planetary radiation belts. Benefiting from their inherent characteristics of low dark current and strong immunity to single-event effects, SiC sensors exhibit distinct application advantages in deep-space radiation monitoring and spacecraft health management.

A conceptual design for a high-precision proton beam monitor system intended for space applications was proposed by He et al., based on the China Spallation Neutron Source (CSNS) 1.6 GeV proton beam. The system uses a 4H-SiC PiN sensor array with front-end electronics, readout system, and mechanical system to enable real-time monitoring of proton energy spectra and flux by measuring energy deposition. The charge collection of SiC PiN sensors after proton radiation was studied using 80 MeV proton beams for continuous running. Monte Carlo simulations indicate an energy resolution of 1.5% for 10 MeV protons, a time response better than 1 ns, and an improvement in detection efficiency for low-energy protons (<1 MeV) by approximately one order of magnitude compared with conventional silicon sensors. The uncertainty of proton beam fluence is below 1% in the beam monitor system [59].

Monte Carlo-based calculations of the Linear Energy Transfer (LET) of SiC for protons, α-particles, and heavy ions of various energies were performed by Xiao et al. using Geant4 simulations. The results showed that proton LET exhibits significant oscillations at low incident energies, gradually decreasing exponentially after 101 MeV. Alpha particles have an LET peak near 1 MeV, while beta particles show an exponential decrease. For different incident angles, the average LET of protons in the low-energy region gradually increases with incident angles, while the effect on alpha particle LET distribution across the full spectrum is not significant. These results provide fundamental data for understanding the energy deposition process and LET distribution in SiC devices under single-particle interactions, which are essential for radiation response modeling of SiC sensors in deep-space exploration [60].

**Figure 5:**
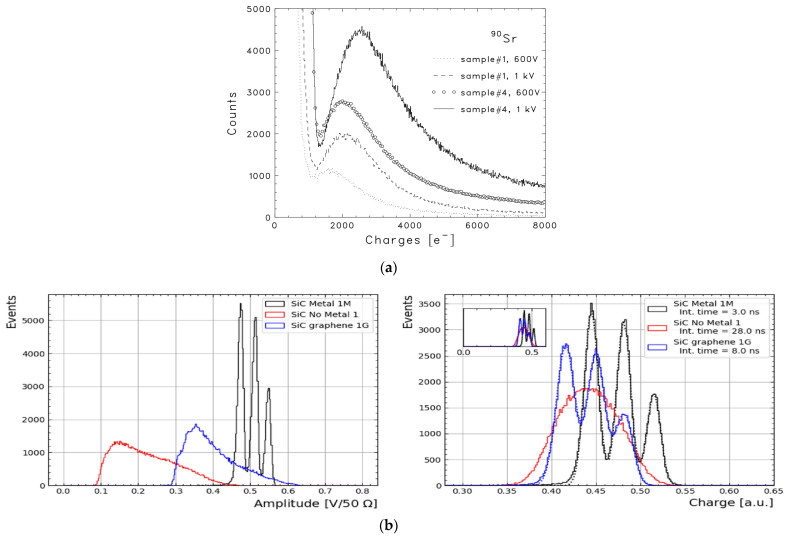
Response characteristics of SiC sensors under proton, alpha particle and heavy ion irradiation: (**a**) Charge response spectrum of SiC p + n diode under equivalent proton (MIP β-ray) irradiation. Reprinted from Ref. [61]. (**b**) Amplitude and charge distribution of SiC detectors under alpha particle (heavy ion) irradiation. Reprinted from Ref. [64].

As shown in Figure 5a, Kishishita et al. reported on SiC p-n junction diodes with a high blocking voltage over 3 kV for beam-monitoring applications. Unlike Schottky barrier types, which have been primarily developed due to simpler fabrication, p-n junction structures are advantageous due to lower sensitivity to surface defects, providing an ideal condition to investigate the effects of bulk crystal defects on radiation sensor characteristics. The p-n diodes were designed with a device simulator and fabricated on 4-inch 4H-SiC wafers with epitaxial layers grown on n-type substrates having a doping concentration of approximately 5 × 10^14^ cm^−3^ and an average thickness of 52 μm. Although fabricated p-n diodes exhibited relatively large leakage currents, they still showed a clear peak of the Landau distribution in charge spectra, suggesting their practical availability as minimum-ionizing-particle detectors. The estimated electron-hole pair creation energy is consistent with published studies [61]. Additionally, Kishishita et al. developed a hybrid SiC pixel detector for charged-particle beam monitors in high-energy physics experiments. By integrating a SiC PiN diode array with a dedicated readout chip, position-resolved detection of heavy ions and protons was achieved. The image obtained with ^90^Sr provided proof of single minimum-ionizing-particle detection capability with all pixels [62]. The design of SiC monolithic active pixel radiation sensors was discussed by Onder et al., who took the first step toward MAPS implementation in SiC-CMOS technology. Using the Fraunhofer IISB 2 μm SiC-CMOS technology, they designed a charge-sensitive amplifier circuit as the first stage in the electronic read-out chain of a MAPS. Circuit simulations showed that an equivalent noise charge of 107 e^−^ is attainable for a sensor capacitance of 1 pF, though the attained bandwidth of 31 kHz was limited by the large size of transistors currently available in SiC-CMOS technology. The authors proposed using 3D integration to vertically stack the sensor and readout circuits, which can effectively reduce power consumption while increasing the fill factor. While SiC-CMOS technology is still in its infancy, with increasing integration density, SiC-MAPS can become a feasible and interesting alternative to their silicon counterparts [63].

High-energy heavy ions in cosmic rays pose a particularly serious single-event effect threat to aerospace electronics. As shown in Figure 5b, the charge-collection characteristics of SiC sensors with an epitaxially grown graphene layer (EG) that substitutes the metallic contact were investigated by Paz et al. To isolate the effect of graphene on charge collection, samples without graphene were produced in parallel. Using a radioactive source and the transient current technique (TCT), which allows for position-dependent signal formation analysis, the study showed that the EG-SiC sensor demonstrates charge-collection capability after signal integration close to that of a fully metallized sensor. The TCT studies revealed uniform charge collection across the active region, as well as an increase in pulse amplitude of up to 40–90% in samples containing no metallic contact, proving that the presence of graphene benefits device performance. The graphene layer effectively suppressed the plasma shielding effect around the heavy ion track, thereby increasing charge-collection speed, and the technology is viable for radiation detection as an alternative to metal [64]. This improvement is of great importance for enhancing the time resolution of real-time single-event monitoring.

For long-duration deep-space missions, the core of radiation dose monitoring lies in the cumulative dose response of the sensor. Depletion depth measurements on novel large-area p-n junction SiC detectors were performed by Spatafora et al. using the ion-beam-induced charge technique with a proton microprobe. The study covered four SiC devices, using proton beams over the 1.26–6.00 MeV incident energy range to probe the active area and depletion depth of each device. The results showed that the square-root relationship between depletion depth and reverse bias remained linear after irradiation, but the maximum depletion voltage increased with neutron fluence. It was found that it is possible to fully deplete the devices provided that the epitaxial layer is properly grown on the substrate. This suggests that the operating bias should be dynamically adjusted according to the cumulative radiation dose [65]. A SiC Timepix3 sensor was used by Novák et al. to perform quantum imaging detection and energy-spectrum tracking of charged particles. The SiC sensor was bump-bonded onto a Timepix3 detector and operated as a compact radiation camera, MiniPIX-Timepix3, with integrated readout electronics. Calibration measurements were conducted with mono-energetic proton beams at energies of 13, 22, 31, 100, and 226 MeV. High-resolution pattern recognition analysis and single-particle spectral tracking were used for detailed inspection of the sensor response. Results included distributions of deposited energy and LET spectra, with examination of the spatial uniformity of the pixelated detector response. The system successfully distinguished protons, α-particles, and heavy ions in a mixed-field simulation of the space radiation environment, with good energy resolution. As a radiation-hard material that can operate at elevated temperatures up to several hundred degrees Celsius, SiC is more suitable for harsh radiation environments compared to conventional Si sensors [66].

### 3.2. Neutron Monitoring in Nuclear Reactors and Fusion Devices

Neutron monitoring within reactor cores and fusion devices places extremely stringent requirements on material radiation hardness and real-time response performance. SiC neutron sensors, which do not require additional moderators, are capable of operating under high-temperature conditions and exhibit gamma-ray insensitivity. Thus, they have developed into a promising alternative to conventional fission chambers and ^3^He tubes.

As reviewed by Ruddy et al., SiC is an ideal material for solid-state nuclear radiation detectors operating in high-temperature, high-radiation environments such as nuclear reactor measurement locations and radioactive waste dismantling operations. The wide bandgap of SiC (3.27 eV) allows low-noise measurements at temperatures up to 700 °C, with thermally induced charge-carrier concentrations at this temperature being approximately four orders of magnitude lower than those in silicon at room temperature. On the other hand, radiation-induced defects can also be exploited for specific sensing scenarios, for example, utilizing nuclear reaction products in neutron detection to generate measurable electrical signals [6,7]. A systematic summary of SiC neutron sensor progress in fission reactor environments indicated that sensors based on ^10^B or ^6^Li conversion layers can achieve thermal neutron detection efficiencies of 5–8%, while the efficiency for fast neutrons is about 0.1–0.5%, relying mainly on elastic scattering of fast neutrons with SiC.

**Figure 6:**
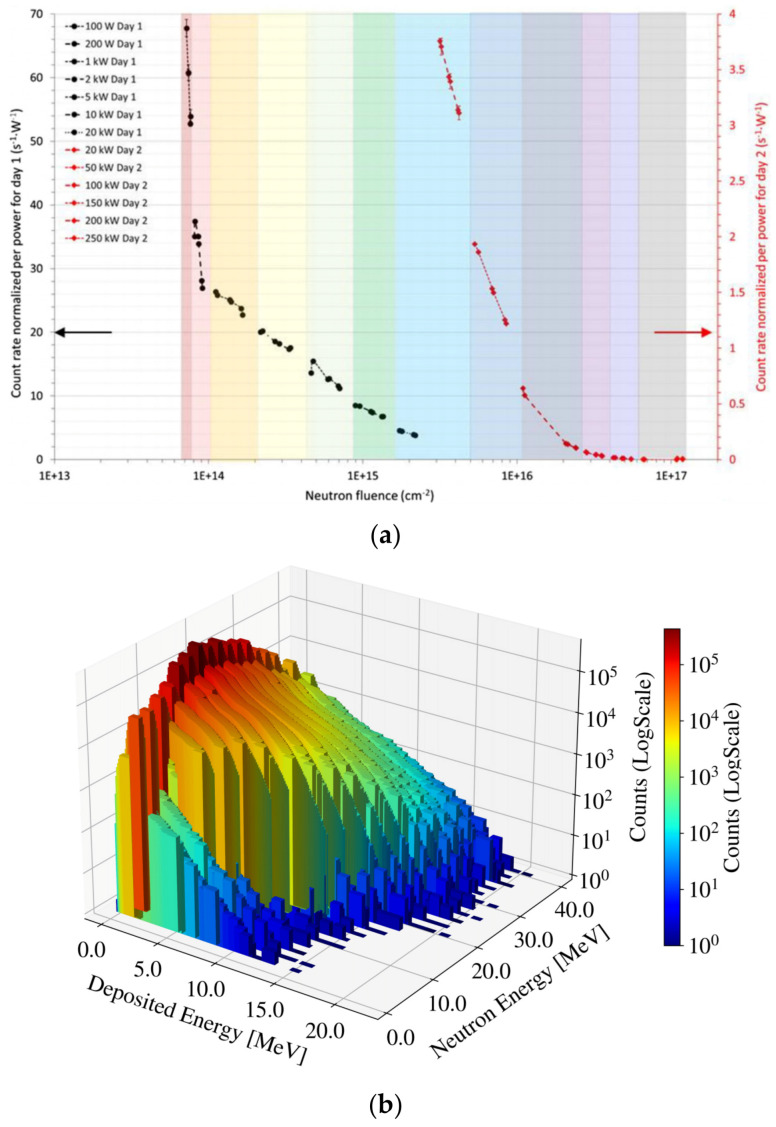
Typical response characteristics of SiC neutron sensors in fission and fusion environments: (**a**) Normalized count rate degradation of a 4H-SiC P^+^N diode with neutron fluence in a fission reactor core. Reprinted from Ref. [67]. (**b**) Experimental response matrix of a 4H-SiC sensor in a multi-energy fast neutron field for fusion application. Reprinted from Ref. [69].

As shown in Figure 6a, at the JSI TRIGA Mark II research reactor, 4H-SiC p-n junction diodes were placed in-core and subjected to continuous neutron irradiation for 300 h by Valero et al. Two types of diodes were used—one with a neutron converter layer of Boron-10 for thermal neutron detection, and another without—to discriminate thermal and fast neutrons by studying the ^10^B reaction versus scattering. Using various pulse-shape analyses and count-rate studies, the influence of bias voltage, the neutron converter layer, and neutron fluence on detector performance was determined. The highest neutron flux and fluence achieved were 1.2 × 10^13^ cm^−2^·s^−1^ and 1.2 × 10^17^ cm^−2^, respectively. The results showed that although dark current rose, the signal-to-noise ratio remained high enough to clearly resolve reactor power changes. The sensor exhibited no signs of packaging failure at a core temperature of 300 °C, whereas under the same conditions, the metal contacts of silicon sensors had already degraded after 100 h [67]. Design considerations for boron-diffused and implanted 4H-SiC epitaxial neutron sensors were further discussed by Ruddy et al. Thermal-neutron detectors based on 4H-SiC semiconductor and ^10^B converter reactions have many advantages for neutron dosimetry and monitoring applications, being capable of stable operation in elevated-temperature environments up to 700 °C for extended periods. The recent development of SiC detectors incorporating ^10^B, achieved by ion implantation or diffusion, offers interesting application-specific design possibilities. It was noted that implanted sensors offer better energy resolution while diffused sensors have higher long-term stability [68].

As shown in Figure 6b, for diagnostics of 14 MeV neutrons in fusion devices, Potiron et al. developed a SiC-based detector system for fast-neutron measurements in multi-energy fields. The study used 4H-SiC diodes with a 60 µm p-n junction that collect charge carriers generated by neutron reaction products and gammas. The calibration of 4H-SiC response functions was performed using Time-of-Flight measurements at the Neutron For Science facility of GANIL, which allowed the construction of a response matrix. The system exploits the differential response of SiC sensors to neutrons of different energies, combined with unfolding algorithms to reconstruct the neutron energy spectrum. In tests at the JET tokamak, the system successfully measured the 14 MeV neutron flux from deuterium–tritium fusion, with results showing good consistency with fission-chamber reference data. The presented results provide a response matrix with sufficient energy channels for neutron spectrum unfolding [69]. However, the current energy resolution is still lower than that of diamond sensors, owing to statistical fluctuations in carrier transport within SiC, and further improvements in material purity and device structure are needed.

Recent progress in high-temperature radiation-hard neutron sensors was reviewed by Wang et al., comparing wide-bandgap materials such as 4H-SiC, diamond, and GaN for fusion neutron diagnostics. The review focused on four different types of neutron detectors: 4H-SiC, diamond detectors, high-temperature fission chambers, and self-powered neutron detectors, analyzing key technological aspects such as their high-temperature and radiation resistance, compact size, and high sensitivity. It was noted that SiC holds significant overall advantages in maturity and cost compared to other wide-bandgap materials, making it a promising candidate for neutron flux measurement in Generation IV nuclear reactor cores, where conventional sensors face considerable challenges due to performance degradation and signal distortion under high-temperature and intense-radiation conditions [70].

In the mixed gamma/n radiation field of nuclear reactors, Pérez et al. achieved pulse shape discrimination between thermal neutrons and gamma rays by placing lead shielding layers of different thicknesses in front of SiC p-n diodes. The SiC diode active detection layer is less than 30 µm thick and provides excellent gamma rejection (5 × 10^−8^), allowing discrimination of neutron-induced events in mixed radiation fields. Experimental tests on a TrueBeam radiotherapy LINAC demonstrated a thermal neutron detection efficiency of (4.32 ± 0.02)% for a (50 ± 10) µm thick ^6^LiF neutron converter, with good linearity and no saturation or dead time effects at dose rates from 100 to 600 MU/min. The experiments revealed that SiC sensors are about 20 times less sensitive to gamma rays than to neutrons, enabling neutron signal extraction without complex electronic discrimination. However, when the gamma dose rate exceeds 10 Gy/s, the accumulated charge signal becomes distorted due to pulse pile-up, limiting its use in accident-condition reactor monitoring [71].

The radiation response of large-area 4H-SiC SBDs was studied by Bernat et al., who compared two diode areas: 1 mm × 1 mm and 5 mm × 5 mm. Using ^6^LiF and ^10^B_4_C films placed on top of the diodes as thermal neutron converters, a thermal neutron efficiency of 5.02% was achieved with a ^6^LiF converter, one of the highest efficiencies reported to date. A temperature-dependent radiation response to alpha particles was presented. Their sensitivity to gamma rays increased with bias but saturated above 100 V, a feature that can be exploited to optimize discrimination parameters in mixed fields. Neutron irradiations were performed in a JSI TRIGA dry chamber, and an Am-241 wide-area alpha source was used for testing the alpha response [72].

Beyond neutron monitoring, SiC sensors also show promise for fission-product monitoring and fuel-failure diagnosis. High-resolution 4H-SiC epitaxial radiation sensors for reactor dosimetry were reported by Mandal et al., summarizing the prospects of Schottky barrier radiation detectors fabricated on highly crystalline, low-defect detector-grade n-type 4H-SiC epitaxial layers with thicknesses ranging from 20 to 250 μm for harsh-environment applications. Characterization of crucial factors limiting energy resolution, such as charge-trap centers, using thermally stimulated transient techniques, was summarized. An energy resolution of 1.2% for fission products was achieved, enabling the distinction of characteristic gamma rays from different fission nuclides. The effect of neutron fluence on detector performance was also discussed [73]. A comparison of 4H-SiC radiation sensors based on PN junctions and Schottky contacts was performed by Kurucová et al. Current-voltage measurements revealed lower leakage current in Schottky-type detectors, resulting in reduced detector noise. Alpha spectra demonstrated better energy resolution for Schottky-contact detectors. Detectors with intermediate epitaxial layer thicknesses (50–60 μm) exhibited optimal performance, characterized by low leakage current, moderate depletion voltage, and efficient charge collection. The study indicated that PN junction sensors have lower leakage current at high temperatures, whereas Schottky sensors respond faster, making them more suitable for transient monitoring. The findings underscore the technological benefits of Schottky contacts and the necessity of optimized epitaxial layer design [74].

In a broader discussion of wide-bandgap semiconductor radiation detectors [2], SiC was systematically compared with GaN and diamond, highlighting that SiC offers the most favorable balance of sensitivity, response speed, radiation hardness, and cost.

### 3.3. Ultra-High-Dose-Rate Radiation Monitoring in FLASH Radiotherapy

FLASH radiotherapy is an emerging cancer treatment modality that uses ultra-high-dose-rate (≥40 Gy/s) radiation beams delivered within milliseconds to ablate tumors while concurrently reducing radiation damage to adjacent normal tissues. This novel therapeutic modality imposes unprecedented performance requirements on the response speed, dose linearity, and radiation hardness of dose monitoring sensors. Benefiting from their rapid response characteristics and high carrier-saturation drift velocity, SiC-based sensors have become a prominent research hotspot in this field.

**Figure 7:**
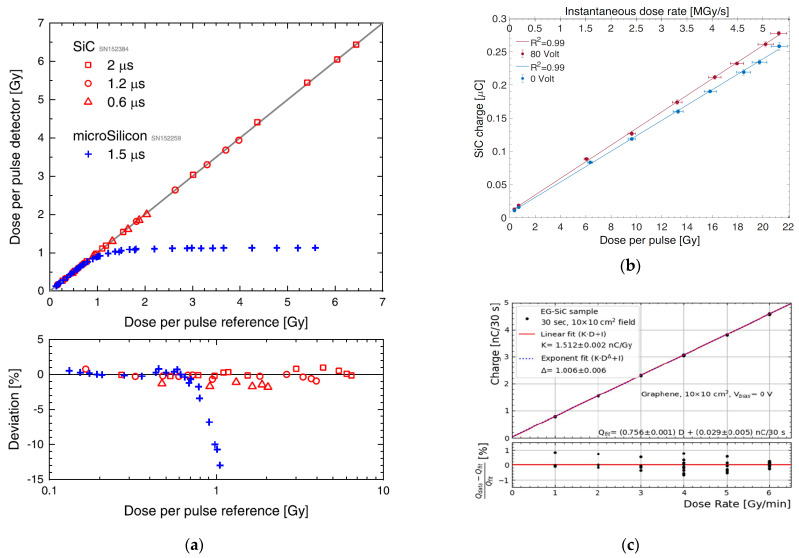
SiC Detectors Under Ultra-High-Dose-Rate Radiation: (**a**) Signal response of the SiC diode and a microSilicon diode with 20 MeV electrons. Reprinted from Ref. [75]. (**b**) Dose-per-pulse linear response of SiC detectors under 0 V and 80 V bias voltages. Reprinted from Ref. [77]. (**c**) Integrated charge over 30 s obtained with the epoxy-covered “Graphene” sample with a Vbias = 0 V, with a 10 × 10 cm^2^ field and 6 MV energy. Reprinted from Ref. [80].

As shown in Figure 7a, the first performance characterization of SiC diode dosimeters for FLASH radiotherapy was reported by Fleta et al. The circular SiC PiN diodes were fabricated in 3 μm epitaxial 4H-SiC and characterized in PTB’s ultra-high-dose-rate reference pulsed electron beam. The SiC diode was operated without an external bias voltage. At electron beam dose rates as high as 1000 Gy/s, the dose response maintained linearity better than 99% up to at least 11 Gy per pulse and 4 MGy/s, with tolerable deviation for relative dosimetry (<3%), whereas commercial silicon diodes already showed obvious superlinear behavior at 100 Gy/s. The sensitivity reduction after 100 kGy accumulated dose was <2%, and the measured temperature coefficient was (−0.079 ± 0.005)%/°C. The SiC diode followed the temporal structure of the 20 MeV electron beam, even for irregular pulse structures, demonstrating its suitability for relative dosimetry in ultra-high-dose-rate pulsed electron beams [75].

Novel SiC sensors designed for ultra-high-dose-rate beams were further developed by Okpuye et al., who investigated the feasibility of using a new generation of SiC detectors for instantaneous dose-rate measurement of UHDR electron beams. The experimental investigation was conducted with the ElectronFLASH linac developed by the SIT Sordina company, capable of accelerating 7- and 9-MeV electron pulsed beams at FLASH regimes. The signals produced in SiC detectors were acquired and compared with signals detected by the monitoring system currently mounted along the LINAC (two AC current transformers). Electrode shape optimization greatly reduced dose-rate dependence. The test demonstrated the capability of the developed SiC detector to measure single pulse duration and waveform with high time resolution and accuracy [76].

A systematic characterization of novel SiC sensors in ultra-high-dose-rate electron and proton beams was carried out by Milluzzo et al. [77,78]. As shown in Figure 7b, a systematic characterization of novel SiC sensors in ultra-high-dose-rate electron beams was carried out by Milluzzo et al. SiC PiN junction detectors with different active areas (ranging from 4.5 to 10 mm^2^) and thicknesses (10–20 μm) were irradiated using 9 MeV UHDR pulsed electron beams accelerated by the ElectronFLASH linac. The linearity of SiC response as a function of delivered dose per pulse was studied under various experimental conditions. Due to extremely high peak currents, an external customized electronic RC circuit was built and used with an electrometer to avoid saturation. The study revealed a linear response for different SiC detectors up to 21 Gy/pulse (corresponding to a maximum instantaneous dose rate of 5.5 MGy/s). The developed devices exhibited a dose-rate-independent response even under extreme instantaneous dose rates and dose-per-pulse values, with dose-rate dependence significantly better than that of diamond sensors. The reliability of these dosimeters with UHDR was also demonstrated without any applied voltage [77]. Moreover, Milluzzo et al. developed an encapsulated waterproof SiC detector for FLASH radiotherapy dosimetry. A 10 μm-thick, 4.5 mm^2^-area SiC detector was embedded in a waterproof, 15 mm-diameter cylindrical housing. This encapsulated version of SiC (eSiC) allows measurement of dose in reference conditions and dose profiles in liquid/solid-water phantoms for high-accuracy dosimetry QA procedures. Using UHDR 9 MeV electron beams, the linearity of charge response as a function of dose per pulse from 1.8 Gy/pulse to about 12 Gy/pulse (IDR 3 MGy/s) was confirmed. With UHDR 228 MeV proton beams, response independence of total delivered dose (1–30 Gy) and average dose rate (50–530 Gy/s) was found. The depth-dose distribution measured with eSiC in a liquid-water phantom was successfully compared with that of a reference chamber. They found that SiC sensors exhibit only a small dose-rate dependence, significantly better than that of diamond sensors. This eSiC prototype can be used to accurately perform reference and relative dosimetry with UHDR electron and proton beams, supporting clinical translation of FLASH radiotherapy [78]. As reviewed by Petringa et al., accurate dosimetry is crucial in radiotherapy and particle therapy to ensure prescribed doses are delivered to tumors while minimizing damage to healthy tissue. SiC, a wide-bandgap semiconductor, has emerged as a promising material for next-generation radiation detectors, showing excellent linearity, radiation tolerance, and the potential to complement or outperform conventional dosimeters. The review highlighted SiC’s role in dosimetry for photon, electron, proton, and carbon-ion beams, including FLASH ultra-high-dose-rate radiotherapy, and surveyed its use across various detector architectures. Ongoing developments and multidisciplinary research are expected to address remaining challenges and pave the way for SiC’s integration into clinical dosimetry [79].

As shown in Figure 7c, Paz et al. explored the use of epitaxially grown graphene as an alternative to metallic contacts for SiC radiation detectors. The first prototypes of SiC diodes with epitaxial graphene contacts were produced at IMB-CNM for radiation detection, along with reference devices. By reducing the amount of metal over the active area, unwanted effects from secondary interactions, which can affect measurement accuracy, can be diminished, essential to meet medical standards of precision. To characterize the feasibility of the technology for medical applications, the dose-rate linearity of the SiC device with graphene was measured in a radiotherapy Linac in the dose rate range of 1–6 Gy/min. The response was compared to that observed on devices with similar geometries reported elsewhere and in a laboratory X-ray tube against a device with a fully metallized active region. The graphene-enhanced SiC detectors with contacts demonstrated excellent dose-rate linearity [80].

López Paz et al. proposed SiC-based diodes as a cost-effective alternative to diamond detectors for dosimetry in UHDR proton beams. Two new SiC diodes designed and fabricated at IMB-CNM were exposed to 20 μs proton low-energy pulses with up to 25 Gy per pulse. The single diode showed good dose-rate linearity with no indication of saturation, even at the highest DPP of 25 Gy, after overexposure to 52 kGy of 7-MeV protons (although its response decreased to 31% of the initial value). A 2 × 2 dosimeter matrix mounted on a motorized stage was used as a proof-of-concept for a large-array dose monitor, with beam profiles observed by the pixelated detector consistent with reference measurements. The full-width half-maximums of the pulses observed by the pixels showed a good correlation with the pulse width of the beam. The SiC detectors were able to withstand and accurately measure dose under FLASH-compatible beam characteristics even for low-energy protons (7 MeV) [81]. Knopf et al. presented a beam-detection setup based on a SiC sensor and a monolithic microwave integrated circuit, capable of detecting single particles with a full-width-at-half-maximum pulse duration of 500 ps. At the MedAustron ion-therapy center, they characterized the spill structure of proton and carbon-ion beams delivered to the irradiation room beyond the timescale of the maximum ion revolution frequency in the synchrotron. The resulting data offer valuable insights into beam intensity at small time scales and demonstrate the capabilities of SiC-based systems for high-flux beam monitoring, which is particularly relevant for characterizing the delivered spill structure of medical proton and carbon-ion beams [82].

Beyond the sensor itself, beam imaging is another important direction for FLASH radiotherapy diagnostics. A SiC Timepix3 sensor was used to perform two-dimensional dose distribution measurements of FLASH electron beams, achieving a spatial resolution of 55 μm and a single-frame dynamic range exceeding 10^5^, capable of simultaneously meeting the needs for low-dose imaging and high-dose-rate monitoring. This capability is of great significance for real-time verification of target localization and normal tissue sparing.

## 4. Material and Device Engineering Strategies for Enhancing Radiation Sensing Performance

The main device structures for SiC radiation detection include SBDs, p-n/PiN junctions, metal-oxide-semiconductor (MOS) capacitors, and Low-Gain Avalanche Detectors (LGADs). SBDs offer simple fabrication and fast response but suffer from higher leakage current and surface sensitivity. The PiN diodes provide lower leakage current and better high-temperature stability but require thicker epitaxial layers and higher operating voltages. MOS structures achieve superior energy resolution and radiation hardness due to reduced surface leakage, but involve more complex processing. LGADs incorporate an internal gain layer to amplify weak signals, improving signal-to-noise ratio, though gain degrades under high-fluence irradiation.

### 4.1. Low-Gain Avalanche Detector Structure

Traditional silicon carbide (SiC) PiN detectors exhibit limited internal signal amplification capability, which frequently results in an inadequate signal-to-noise ratio when detecting low-ionization-density radiation, including low-energy X-rays and thermal neutrons. The Low-Gain Avalanche Detector (LGAD) structure addresses this limitation effectively by integrating an internal gain layer into the device, which enables in situ signal amplification while retaining a fast temporal response.

**Figure 8:**
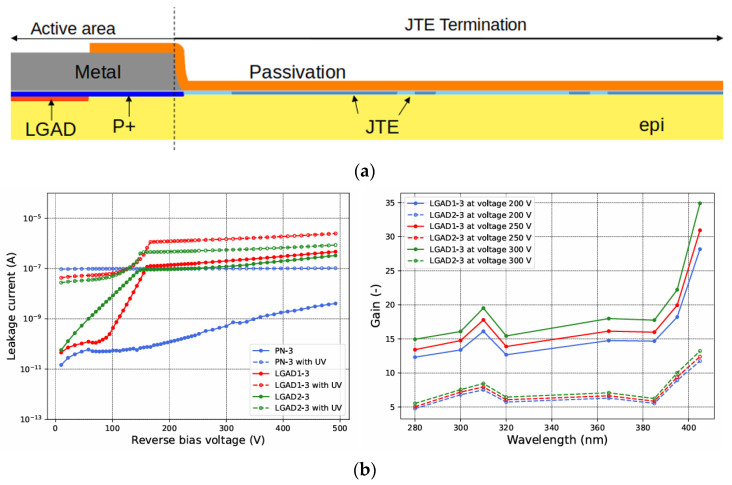
Gain layer design and gain variation characteristics of 4H-SiC LGADs: (**a**) Illustration of the 4H-SiC LGAD cut view. (**b**) Response to UV light: Leakage current of the measured sensors [83].

As shown in Figure 8, the fabrication and performance evaluation of first-generation 4H-SiC Low-Gain Avalanche Detectors produced by onsemi were reported by Novotný et al. The devices are specifically designed and optimized for fabrication on n-type substrate/epi wafers with the gain layer implanted approximately 1 μm below the surface. By implementing a charge multiplication layer to address the lower signals generated by minimum ionizing particles in 4H-SiC compared to standard silicon, due to its higher bandgap energy, an intrinsic gain of the device was achieved. The measured properties of these detectors, including temperature stability and the effectiveness of the internal gain layer in improving signal generation, align well with predictions from extensive TCAD simulation studies. An avalanche gain of about 20 was achieved, while the dark current increased only about fivefold [83].

Zhao et al. fabricated a 4H-SiC Low-Gain Avalanche Detector named SICAR and reported its electrical characteristics and charge-collection performance. By optimizing the fabrication process, the leakage current of the detector was reduced by four orders of magnitude. Using a multi-step epitaxial growth technique, a steeper doping profile was produced, and gain uniformity improved from ±30% to ±10%. Experimental results confirmed that this 4H-SiC LGAD exhibits a distinct gain structure, with a gain factor of about 3 at 350 V for 5.54 MeV alpha particles. After high-fluence neutron irradiation, the optimal operating voltage shifted to higher values by about 50 V, but the gain factor could still be maintained. This study provides a novel 4H-SiC LGAD radiation detector for applications in high-energy particle physics, demonstrating the robustness of this structure in radiation environments [84]. TCAD simulations were employed by Onder et al. to design and optimize a 30-μm-thick 4H-SiC Low-Gain Avalanche Diode (LGAD) for high-voltage operation. A 2.4-μm-thick epitaxially grown gain layer enables controlled internal amplification up to 1 kV reverse bias while maintaining full depletion below 500 V. Electrical characteristics, including I-V, C-V, and gain behavior, were simulated in Synopsys Sentaurus TCAD using a quasi-1D geometry and verified across process-related variations in gain-layer parameters. To ensure high-voltage stability and proper edge termination, a guard structure combining deep-etched trenches and deep p+ junction–termination–extension implants was designed. TCAD simulations varying the guard structure dimensions yielded an optimized design with a breakdown voltage above 2.4 kV, showing that the trench isolation structure effectively suppresses crosstalk between pixels, reducing charge sharing between adjacent pixels from 15% to below 3% [85].

An exploration of next-generation 4H-SiC LGADs by Svihra et al. presented the design, production, and initial testing of newly developed 4H-SiC Low-Gain Avalanche Detectors fabricated by onsemi. The evaluation includes performance metrics such as the internal gain layer’s efficiency in enhancing signal generation. Due to the wider bandgap of 4H-SiC compared to standard silicon and the difficulty of producing high-quality layers thicker than 50 μm, an internal charge-multiplication layer needs to be introduced. Initial transient current technique and laboratory test results demonstrate fast charge collection and uniform multiplication across multiple samples produced on a single wafer. The devices are optimized for N-type substrate and epi wafer configurations, showing promise for detecting both low-energy X-rays and for withstanding direct high-fluence proton beam irradiation [86]. It has been pointed out that the LGAD structure is a key development direction for SiC particle physics sensors, and large-scale, pixelated LGAD devices are expected to reach engineering applications within the coming years [87].

Simulation studies by Tan et al. on 3D electrode structures investigated a novel 3D 4H-SiC detector to meet high-radiation challenges for detectors in future high-energy physics. The study found that charge-collection time can be reduced to sub-nanosecond levels, with greater tolerance to radiation-induced trapping effects. This structure can serve as a complementary approach to LGADs by improving the signal-to-noise ratio through shortened carrier drift distance without avalanche gain. The rough time resolution of the 3D 4H-SiC detector was estimated, and simulation parameters can serve as guidelines for 3D 4H-SiC detector design and optimization [88]. Yang et al. determined the time resolution of 100 μm 4H-SiC PiN detectors fabricated by Nanjing University. The time response to β-particles from a ^90^Sr source was investigated for minimum-ionizing-particle detection. The influence of different reverse voltages (corresponding to different carrier velocities and device sizes) was studied, as well as the correlation with detector capacitance. A time resolution of (94 ± 1) ps was determined for the 100 μm 4H-SiC PiN detector. A fast simulation software called RASER was developed and validated by comparing waveforms from simulated and measured data. The simulated time resolution was (73 ± 1) ps after considering the intrinsic leading contributions of the detector to time resolution. This performance level approaches that of LGADs but without requiring a high bias voltage [89]. Furthermore, Jiang et al. fabricated a graphene-optimized 4H-SiC p-i-n detector that not only meets detection requirements for low-penetration particles, UV light, and medical dosimetry but also shortens signal rise time. The effective doping concentration of the lightly doped 4H-SiC epitaxial layer is about 4.5 × 10^13^ cm^−3^, approaching the limit of the lowest doping level by SiC epitaxial growth. The rise time of the graphene-optimized ring electrode detector was reduced by 24% at 200 V compared to conventional ring electrode detectors, corresponding to a reduction from 2.1 ns to 1.2 ns. The CCE of graphene-optimized 4H-SiC p-i-n detectors was 99.22%, an increase of approximately 12%. When graphene was irradiated with an 80-MeV proton beam at a fluence of 2.1 × 10^11^ neq/cm^2^, the irradiation had no significant impact on the rise time or its uniformity, proving that graphene has certain radiation resistance. This research may expand the applications of graphene-based 4H-SiC detectors in fields such as low-penetration particle detection, high-energy particle detection, medical dosimetry, and transient current technique measurements [90].

### 4.2. Metal-Oxide-Semiconductor Structure and Heterogeneous Contact Engineering

Owing to its advantages of low leakage current and adjustable depletion region, the MOS structure has gained extensive research attention in the field of radiation detection. The integration of the MOS structure with SiC can effectively inhibit radiation-induced surface leakage current and maintain the signal-to-noise ratio of the detector after cumulative radiation exposure.

The first application of Ni/SiO_2_/n-4H-SiC vertical MOS capacitors for high-resolution radiation detection was reported by Chaudhuri et al. The 100 nm SiO_2_ layer was achieved on the Si face of n-4H-SiC epilayers using dry oxidation in air. By modulating the depletion region depth with the gate voltage, the device achieved an excellent energy resolution of 0.42% for 5.48 MeV alpha particles, outperforming the best high-resolution Schottky barrier detectors (which showed lower energy resolution). The MOS detectors also exhibited a high CCE of 96% at optimized operating bias despite the presence of the oxide layer. A drift-diffusion model applied to the CCE vs. gate bias voltage data revealed a minority (hole) carrier diffusion length of 24 μm. Capacitance-mode deep-level transient spectroscopy scans were performed over the temperature range 84–800 K to identify resolution-limited electrically active defects [91]. Karadavut et al. reported further optimization of vertical Ni/SiO_2_/4H-SiC MOS capacitors for high-resolution radiation detection. The MOS detectors were fabricated on 50-μm-thick n-type 4H-SiC epitaxial layers by growing a silicon dioxide layer thermally prior to contact deposition. Through post-nitridation annealing, the interface state density was reduced from the 10^12^ cm^−2^ eV^−1^ level to the order of 10^11^ cm^−2^ eV^−1^, lowering the sensor dark current density by an order of magnitude. The MOS detectors exhibited leakage currents two orders of magnitude lower than Schottky barrier detectors fabricated on similar epilayers and showed a much higher energy resolution of 0.4% compared to 0.8% for SBDs for 5486-keV alpha particles, although both devices showed 96% CCE. After a cumulative gamma dose of 10 Mrad, the energy resolution degradation of the optimized sensor was less than 0.2%, compared with over 1% for the unoptimized device. The higher resolution of the MOS device is attributed to lower leakage current and differences in defect configurations [92].

**Figure 9:**
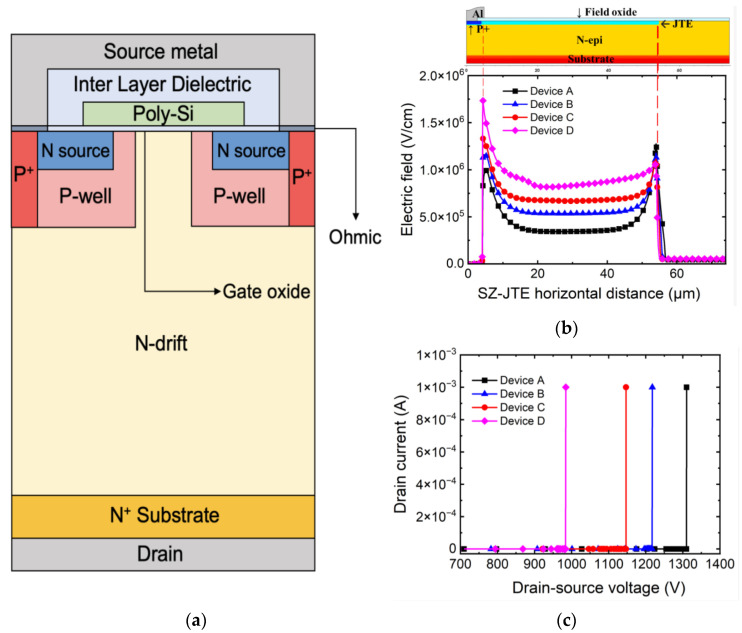
(**a**) A cross-sectional view of a conventional SiC MOSFET. (**b**) The electric field distribution of SZ-JTE with the consideration of gamma irradiation. (**c**) Breakdown characteristics of SZ-JTE with consideration of gamma irradiation. Reprinted from Ref. [93].

As shown in Figure 9, radiation environments such as proton and gamma irradiation can severely affect the electrical characteristics of SiC metal-oxide-semiconductor field-effect transistors (SiC MOSFETs) and may ultimately lead to power system failures under long-term radiation exposure. Kim et al. irradiated a 1.2 kV SiC MOSFET with gamma rays and protons and evaluated the evolution of its electrical characteristics to analyze the degradation mechanisms induced by total ionizing dose (TID). The experimental results showed that the threshold voltage and on-resistance decreased with increasing gamma-ray dose and proton fluence. This degradation was attributed to the accumulation of positive fixed charges in the gate oxide layer, which increased with radiation exposure. Additionally, degradation in breakdown voltage was observed, attributed to charge trapping in the field oxide layer, altering the depletion curvature at the edge of the termination region. These findings provide insights into understanding the TID-induced degradation mechanisms in SiC MOSFETs under different radiation conditions [93]. Ji et al. systematically evaluated the effects of introducing a SiO_2_ dielectric layer to form a MOS structure on the electrical properties, performance, and radiation hardness of 4H-SiC α-particle detectors, comparing with metal/semiconductor (MS) structures. It was confirmed that the MOS structure can effectively enhance the radiation hardness of SiC sensors: after growing a high-quality SiO_2_ layer on the SiC surface, the reverse leakage current and signal-to-noise ratio at high voltages were improved. The density of proton-irradiation-induced surface defects was reduced by approximately two orders of magnitude, and the sensor dark current stability improved about fivefold, an effect attributed to the passivation of surface dangling bonds and the blocking of radiation-generated charges by the oxide layer. The MOS structure exhibited much higher radiation hardness, as the energy resolution degradation of α-particle detectors with MOS and MS structures after gamma irradiation was 0.59% and 2.87%, respectively [94].

Graphene has also been introduced as a contact layer in SiC sensors [64,80], taking advantage of its high carrier mobility and chemical inertness to suppress radiation-induced contact degradation. Zhang et al. investigated the electrical output characteristics and noise performance of SiC-based junction field-effect transistors (JFETs) at high temperatures. Due to the intrinsic properties of SiC, such as its wide bandgap and incomplete ionization, simply substituting silicon with SiC in an identical structure does not achieve optimal results. The study systematically explored how JFET structural design influences device performance by comparing electrical characteristics and output noise of devices with varying channel parameters across a range of temperatures. Results highlighted the effects of channel doping concentration and channel height on transconductance and equivalent output noise under high-temperature conditions. The experimental fabrication and performance evaluation of SiC-JFETs specifically designed for front-end circuits in nuclear electronics showed that a preamplifier employing a JFET input stage reduces the equivalent noise charge of the SiC sensor by approximately 30%, making it particularly suitable for weak-signal radiation detection. These findings provide a foundation for advancing SiC-JFET-based front-end electronics in nuclear detection systems [95].

In heterogeneous integration, Meguro et al. fabricated a radiation-hard CMOS image sensor by hybrid integration of a Si photodiode and 4H-SiC MOSFETs using direct heterogeneous bonding. The hybrid pixel device consists of one Si photodiode and three 4H-SiC nMOSFETs. During fabrication, the SOI substrate was directly bonded on 4H-SiC substrate via SiO_2_. After bonding, the base silicon substrate and buried oxide were removed by TMAH wet etching. Using this SOI-Si/4H-SiC substrate, the SOI-Si photodiodes and 4H-SiC nMOSFETs were integrated on the same substrate. This hybrid pixel structure combines the high quantum efficiency of Si with the radiation-hard readout circuit of SiC. After MGy-level gamma irradiation, the increase in dark current was only one-tenth that of an all-Si scheme, opening a new path for radiation imaging sensors with ultra-large dynamic range. A response of the SOI-Si/4H-SiC hybrid pixel device to light illumination was successfully demonstrated [96]. Pascu et al. developed a wide-temperature-range temperature sensor based on a 4H-SiC Schottky diode, covering 60 K to 700 K (currently the widest range reported) with a sensitivity of 2.32 mV/K at 300 K for CTAT operation, and satisfactory linearity (R^2^ reaching 99.80%) even down to 60 K. The structure’s layout places two identical diodes in close, symmetrical proximity, using a stable and high-barrier Schottky contact based on Ni annealed at 750 °C. Forward measurements in the 60–700 K range indicate nearly identical characteristics for the dual-diodes with only minor inhomogeneity. The PTAT differential version boasts increased linearity up to 99.95%. After high-fluence fast neutron irradiation, the sensitivity changed by less than 2%, demonstrating the potential of SiC temperature sensors in nuclear environments. The lower sensitivity in PTAT mode is compensated by using a high-performing, low-cost readout circuit, leading to a peak of 14.91 mV/K without influencing linearity [97].

### 4.3. Material Growth and Defect Engineering

The crystalline quality of SiC materials directly determines the baseline radiation sensing performance and degradation rate of the resultant devices. The intrinsic radiation hardness of SiC-based radiation sensors can be improved through three key approaches: optimizing the epitaxial growth process, regulating the concentration of intrinsic defects, and introducing buffer layer structures.

Capan comprehensively reviewed the application of n-type 4H-SiC SBDs as radiation detectors, highlighting significant progress achieved over the past decade in SBD fabrication, electrical characterization, and radiation response. SiC, especially the 4H-SiC polytype, possesses a wide bandgap, high critical breakdown electric field, high thermal conductivity, and excellent resistance to displacement damage, making it one of the most promising alternative materials for extreme-environment sensing [2,3]. For fusion reactor applications demanding extreme radiation hardness, the use of high-purity semi-insulating SiC substrates instead of conductive ones has been recommended to reduce radiation-induced leakage current, although carbon vacancy-related defects in such substrates require post-growth annealing at 1600 °C in an argon atmosphere [3].

**Figure 10:**
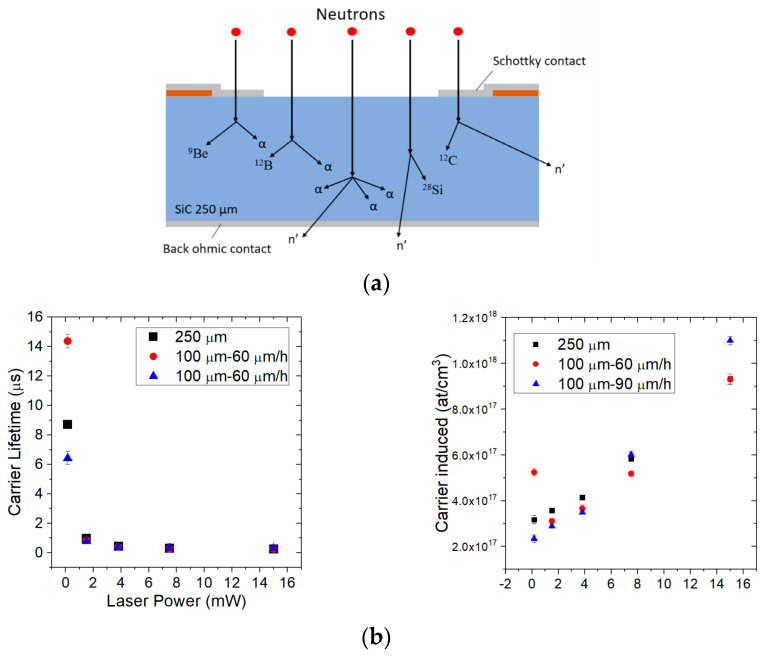
(**a**) Scheme of the common interactions that take place on the epitaxial layer of SiC devices. (**b**) Carrier lifetime of 4H-SiC epilayers with different thicknesses and growth rates. Reprinted from Ref. [98].

As shown in Figure 10, the influence of epitaxial growth conditions on the neutron detection performance of 4H-SiC was systematically studied by Meli et al. The study examined high-growth-rate processes to grow thick epitaxial layers (250 μm) of 4H-SiC, comparing results with samples having 100 μm epitaxial layers grown at two different growth rates (60 and 90 μm/h). Photoluminescence spectroscopy was used for stacking fault defect evaluation, while micro-Raman spectroscopy was used for simultaneous determination of both carrier lifetime and induced carriers in equilibrium. Raman measurements showed that both growth rate and epitaxial layer thickness affect the measured carrier lifetime. Epitaxial layers grown with optimized conditions exhibited the lowest basal-plane dislocation density, and sensors fabricated from this material displayed significantly superior CCE compared with those made from commercial wafers. The evaluation of stacking faults on carrier lifetime as a function of injection level showed that only at low injection is the effect on carrier lifetime low [98]. A monolithic integration process of graphene with SiC radiation sensors was explored by Ugobono et al. as part of the GRACE project, which seeks to deliver a new generation of SiC sensors with graphene-enhanced contacts aimed to be radiation-hard and functional at high temperatures. By directly growing graphene via thermal decomposition on the SiC surface, contamination and damage during graphene transfer were avoided. The work focused on the optimization of electrical contacts, along with electrical characterization and radiation-tolerance assessment of the first sensor prototypes produced. The integrated sensor showed better performance stability than discrete devices in simulated space radiation environments, leveraging the inherent advantages of SiC devices, including low leakage current, low noise levels, high thermal conductivity, and potential radiation hardness for harsh-environment applications such as plasma diagnostic systems in future nuclear fusion reactors or high-energy physics applications [99].

A manufacturing process for SiC-TiC composites was optimized by Ivzhenko et al. using the electroconsolidation method (spark plasma sintering) at a pressure of 45 MPa. The influence of titanium carbide content on physical and mechanical properties was studied. Compared to sintered SiC, composites with 40 mol% TiC showed decreased porosity from ~30% to 0%, increased crack resistance from 2.9 to 6.1 MPa·m^0.5^, and increased hardness from 2.9 to 21.5 GPa. An increase in temperature from 1900 °C to 2000 °C resulted in an approximately 30% rise in composite hardness, while extending sintering time from 30 to 45 min decreased both fracture toughness and hardness. The addition of TiC through electro-consolidation increased flexural strength while maintaining excellent thermal conductivity, offering a new route for large-scale SiC sensor structural components [100].

A comparison of annealing temperatures for recovery of SiC sensors after electron, neutron, and proton irradiation was performed by Rafi et al. Radiation effects in irradiated 4H-SiC p-n junction diodes were investigated by means of electrical characterization, including I-V characteristics measured at temperatures ranging from −50 °C to +200 °C. The stability of radiation-induced effects was evaluated through a series of low-temperature treatments (up to 400 °C). The study found that annealing can effectively eliminate point defects but has a limited effect on defect clusters. While extremely high-temperature annealing can dissociate defect clusters, it may cause metal contact failure. Partial recovery of diode rectification functionality was observed for electron-irradiated devices, and partial recuperation of detectors’ CCE was registered on all irradiated devices. Interestingly, the limited conduction registered for highly irradiated SiC detectors allows their operation in forward bias conditions, which, while providing somewhat lower CCE, shows better energy resolution than in conventional reverse bias operation [101]. This indicates that for applications requiring online annealing recovery, a careful trade-off between annealing temperature and packaging tolerance is necessary.

Liu et al. reviewed the progress of one-dimensional SiC nanomaterials for sensing applications. These materials hold great promise for nanoelectronic devices, sensors, supercapacitors, and catalyst carriers due to their unique electrical, mechanical, and physicochemical properties. Recent progress in their design and fabrication has led to a deep understanding of structural evolution and structure-property correlation. Unique attributes, such as high electron mobility, offer opportunities for designing SiC-based sensors with high sensitivity, which could potentially be extended to radiation sensing applications [102]. Wang et al. reviewed femtosecond laser processing of SiC, a promising semiconductor material that is challenging to machine due to its high hardness, superior thermal conductivity, and chemical inertness. The ultrafast nature of femtosecond lasers enables precise and controlled material removal and modification, making them ideal for SiC processing. The review covered various methodologies, including direct processing, composite processing, modification of the processing environment, and beam shaping, as well as myriad applications arising from applying femtosecond laser processing to SiC. These techniques are particularly relevant for manufacturing SiC-based sensor structures for harsh-environment applications [103]. Jin et al. investigated the surface modification of SiC wafers using atmospheric plasma etching, which is essential for manufacturing semiconductor devices. Due to SiC’s ultra-hardness and remarkable chemical inertness, surface processing faces significant challenges requiring highly efficient, damage-free methods. The study determined optimal processing parameters (input power: 550 W; processing distance: 3.5 mm; Ar/CF4/O2 flow rates: 15 SLM/70 SCCM/20 SCCM) that enable rapid, uniform removal of the wafer surface. These techniques are relevant for preparing high-quality SiC substrates for radiation sensor fabrication [104].

Dietz et al. developed a selective photoelectrochemical (PEC) etching process for 4H-SiC that relies not on high doping but on the electrical depletion of a fabricated diode structure, allowing selective etching of an n-doped substrate wafer versus an undoped epitaxial device layer. This technique overcomes the chemical inertness of SiC that makes complex micromachining difficult, despite its desirable properties for MEMS and harsh-environment sensors. The work enabled the suspension of large (100 × 100 μm) undoped membranes of SiC and demonstrated that using undoped material improves the ensemble spin lifetime, which is particularly relevant for quantum sensing applications [105]. Kulkarni et al. developed a laser-assisted boron doping technique for n-type 4H-SiC using a pulsed Nd:YAG laser with a liquid-phase boron precursor, overcoming the historical challenges of chemical inertness and low dopant diffusivity in SiC. By leveraging a heat-transfer model to optimize laser process parameters, dopant incorporation was achieved while preserving crystalline integrity. The laser-fabricated p-n junction diode demonstrated a reverse-breakdown voltage of 1668 V. This technique offers a new route for fabricating SiC-based sensor structures with tailored doping profiles [106]. Sapienza et al. reported the fabrication of wafer-level vacuum-packaged 3C-SiC resonators obtained from layers grown on <100> and <111> silicon. The resonant microstructures are double-clamped beams encapsulated by glass-silicon anodic bonding using titanium-based vacuum gettering. Open-loop resonance frequency measurements showed Q-factor values up to 292,000 for <100> and 331,000 for <111> substrates, with a maximum vacuum level around 10^−2^ mbar inside encapsulations with a Ti getter. These high-Q resonators are relevant for SiC-based MEMS sensing applications, including those requiring radiation tolerance [107].

Liu et al. developed and validated an accurate interatomic potential for molecular dynamics simulations of 3C-SiC using a deep-learning framework combined with a smooth ZBL-screened nuclear-repulsion potential interpolation. For significant properties of radiation damage, such as defect formation energies and threshold displacement energies, their deep-learning potential gave closer predictions to the DFT criterion than existing analytical potentials. This framework solves the long-standing dilemma that traditional empirical potentials currently applied in 3C-SiC radiation damage simulations give large disparities and are inconsistent with ab initio calculations, enabling a more realistic depiction of primary irradiation damage processes [108]. Yan et al. reviewed molecular dynamics simulation studies of SiC materials for properties, preparation, and performance. SiC materials are widely applied in nuclear materials and semiconductor materials due to their excellent radiation resistance, thermal conductivity, oxidation resistance, and mechanical strength. The review found that the Tersoff potential is the most widely applied potential function for MD simulations of SiC materials. In MD simulations of SiC performance, most studies are related to SiC applications in nuclear energy research, with irradiation damage simulation being the most widely studied topic. This review provides a good reference value for understanding computational materials science methods for multi-level analysis of SiC materials [109]. Li and Liu studied the interplay of irradiation-induced defects with oxygen in 3C-SiC ceramics using systematic first-principles calculations. The formation energy of oxygen at the carbon site (OC) is negative, implying that dissolution of oxygen in the host carbon site is exothermic. The binding strengths of interstitial oxygen and OC with irradiation-induced defects are notably high. The formation energies of oxygen and carbon vacancy clusters decrease approximately linearly and turn negative as the number of oxygen atoms increases, indicating a strong attraction between them. Trapping of oxygen by carbon vacancies may reduce oxygen mobility. The study suggests that irradiation-induced defects may enhance the oxidation of SiC due to strong binding energies, which have important implications for the lifetime assessment of SiC components in nuclear power systems under extreme conditions [110].

## 5. Conclusions

With a wide bandgap of 3.26 eV, high critical breakdown electric field, high thermal conductivity, and exceptional resistance to displacement damage, SiC has emerged as one of the most promising materials for sensing technologies in extreme radiation environments. This review systematically organizes the multidimensional research progress regarding the performance evolution of SiC sensors in radiation environments and elucidates the structure–activity relationships from microscopic defects to macroscopic device responses. It further conducts an in-depth analysis of the key technical challenges encountered in typical application scenarios, including deep-space exploration, nuclear reactor monitoring, fusion device diagnostics, and FLASH radiotherapy.

The effect of radiation on the sensing performance of SiC presents distinct dual characteristics. On the one hand, point defects, defect clusters, and even amorphous regions generated by the interaction between high-energy particles/photons and the SiC lattice act as recombination centers or charge traps, which substantially alter the generation, transport, and recombination processes of charge carriers. This directly induces the degradation of CCE, the attenuation of the piezoresistive coefficient, and the elevation of leakage current. On the other hand, radiation-induced nuclear reaction products—especially the charged particles produced via ^10^B or ^6^Li conversion layers in neutron detection—can be utilized to generate measurable electrical signals. This “defect-as-function” perspective establishes a novel paradigm for sensor design targeting specific radiation scenarios.

The differential effects of various radiation types on the sensing performance of SiC constitute the core analytical framework of this review. Neutron irradiation induces high-density displacement cascade damage via nuclear collisions, resulting in a three-stage degradation characteristic of CCE as neutron fluence increases: the isolated-point-defect-dominated phase, the defect-cluster-formation phase, and the carrier-transport-channel-blockage phase. The effects of proton irradiation exhibit significant energy dependence: low-energy protons, due to their higher nuclear stopping power, generate high-concentration defects in the near-surface region, while high-energy protons are dominated by electronic stopping, with damage concentrated at the end of particle trajectories. Heavy ion irradiation simultaneously induces the generation of high-density electron–hole pairs and severe displacement damage, which is exceptionally destructive to sensor performance; nevertheless, SiC devices still exhibit radiation resistance that is more than two orders of magnitude higher than that of silicon devices. Gamma irradiation primarily causes ionization damage and interface state generation, leading to threshold voltage shifts and switching characteristic degradation, yet SiC bipolar devices can maintain acceptable performance degradation at cumulative doses up to 2 Mrad.

The synergistic effect of high temperature and radiation exposes more complex failure mechanisms. Under high-temperature irradiation, the radiation resistance of SiC is significantly enhanced—the carrier removal rate at 500 °C irradiation is approximately six orders of magnitude lower than that under room-temperature irradiation, which is attributed to the rapid annealing and recombination of primary radiation defects at elevated temperatures. However, the composition of residual defects changes after high-temperature irradiation: the proportion of vacancy-type defects decreases while that of antisite defects increases, which may lead to different long-term stability outcomes compared with room-temperature irradiation. Furthermore, the substantial reduction in the thermal conductivity of SiC (from ~350 W/(m·K) to below 100 W/(m·K)) induced by high-fluence neutron irradiation exacerbates Joule heat accumulation, forming a “radiation–thermal–electrical” positive feedback failure mechanism. This problem is particularly prominent in applications such as fusion reactor diagnostic windows.

In terms of application practice, SiC sensors have exhibited unique advantages in multiple extreme radiation scenarios. In deep-space exploration, their low dark current and immunity to single-event effects make them optimal candidates for space radiation monitoring and spacecraft health management. For in-core neutron monitoring in nuclear reactors, SiC sensors based on ^10^B or ^6^Li conversion layers have achieved continuous in-core operation for more than 300 h without packaging failure, while maintaining a signal-to-noise ratio sufficient to resolve changes in reactor power. In the emerging field of FLASH radiotherapy, SiC sensors maintain linearity better than 99% under ultra-high-dose-rate electron beams up to 1000 Gy/s, achieve sub-nanosecond time resolution, and exhibit cumulative dose tolerance that far exceeds that of conventional silicon sensors.

Considerable progress has been achieved in three directions regarding material and device engineering strategies for improving radiation sensing performance. The LGAD structure realizes internal signal amplification by integrating a gain layer inside the sensor, which effectively improves the signal-to-noise ratio for detecting low-ionization-density radiation while maintaining a fast time response and retaining gain characteristics even after high-fluence irradiation. The MOS structure, via introducing a high-quality SiO_2_ dielectric layer, reduces the surface defect density by approximately two orders of magnitude and improves the dark current stability of the sensor by about five times, providing an effective solution for high-resolution detection in intense radiation environments. At the material level, optimizing epitaxial growth processes to reduce basal-plane dislocation density, regulating intrinsic defect concentrations via high-temperature annealing, and introducing novel contact materials, such as graphene, to suppress irradiation-induced contact degradation are all feasible pathways to improve the radiation hardness of SiC sensors.

Despite these remarkable advances, multiple critical challenges still exist for the application of SiC sensors in extreme radiation environments. First, the long-term reliability under cumulative doses exceeding the MGy level requires systematic verification, especially the temporal evolution of irradiation-induced defects and their cumulative impact on sensing performance. Second, under the coupled condition of high temperature and intense radiation, the thermal mismatch failure at the packaging interface and the degradation of metal contacts are problems that require urgent resolution. Third, for specific applications such as 14 MeV neutron diagnostics in fusion devices, the energy resolution of current SiC sensors still lags behind that of diamond detectors, necessitating breakthroughs in both material purity and device structure. In addition, the performance degradation of signal readout electronics in high-temperature radiation environments remains a technical bottleneck restricting the overall reliability of SiC-based sensing systems.

Prospectively, the development of SiC-based sensors should focus on the following directions: (1) establishing quantitative irradiation damage models covering multiple radiation types, wide fluence ranges, and broad temperature windows to provide theoretical support for sensor lifetime prediction; (2) developing integrated and intelligent SiC sensing systems that achieve the integration of sensing, amplification, and readout via monolithic or heterogeneous integration; (3) exploring novel sensing mechanisms based on radiation defect engineering, and converting the negative effects of radiation damage into enhanced functions for specific signals; (4) promoting experimental validation of novel device architectures, such as 3D electrode structures and super junction structures, to break through the performance limits of conventional planar configurations. With the synergistic advancement of material growth processes, device design, and system integration, SiC sensors are expected to play an irreplaceable role in next-generation deep-space exploration, advanced nuclear energy systems, fusion devices, and precision radiotherapy.

## Data Availability

No new data were created or analyzed in this study. Data sharing is not applicable to this article.

## Abbreviations

The following abbreviations are used in this manuscript:

DLTS	Deep-Level Transient Spectroscopy
LET	Linear Energy Transfer
CCE	Charge-Collection Efficiency
UV-TCT	Ultraviolet Transient Current Technique
CSNS	China Spallation Neutron Source
LGAD	Low-Gain Avalanche Detector
MOS	Metal-Oxide-Semiconductor
